# Early Myeloid Derived Suppressor Cells (eMDSCs) Are Associated With High Donor Myeloid Chimerism Following Haploidentical HSCT for Sickle Cell Disease

**DOI:** 10.3389/fimmu.2021.757279

**Published:** 2021-11-30

**Authors:** Deepali K. Bhat, Purevdorj B. Olkhanud, Arunakumar Gangaplara, Fayaz Seifuddin, Mehdi Pirooznia, Angélique Biancotto, Giovanna Fantoni, Corinne Pittman, Berline Francis, Pradeep K. Dagur, Ankit Saxena, J. Philip McCoy, Ruth M. Pfeiffer, Courtney D. Fitzhugh

**Affiliations:** ^1^ Cellular and Molecular Therapeutics Branch, National Heart, Lung, and Blood Institute (NHLBI), National Institutes of Health (NIH), Bethesda, MD, United States; ^2^ Bioinformatics and Computational Biology Core Facility, National Heart, Lung, and Blood Institute (NHLBI), National Institutes of Health (NIH), Bethesda, MD, United States; ^3^ Center for Human Immunology, Autoimmunity, and Inflammation, National Institutes of Health (NIH), Bethesda, MD, United States; ^4^ Flow Cytometry Core, National Heart, Lung, and Blood Institute (NHLBI), National Institutes of Health (NIH), Bethesda MD, United States; ^5^ Division of Cancer Epidemiology and Genetics, National Cancer Institute, National Institutes of Health (NIH), Bethesda, MD, United States

**Keywords:** donor myeloid chimerism, haploidentical HSCT, Tregs, IL-10, sickle cell disease, early myeloid derived suppressor cells

## Abstract

Haploidentical hematopoietic stem cell transplantation (haplo-HSCT) is a widely available curative option for patients with sickle cell disease (SCD). Our original non-myeloablative haplo-HSCT trial employing post-transplant (PT) cyclophosphamide had a low incidence of GVHD but had high rejection rates. Here, we aimed to evaluate immune reconstitution following haplo-HSCT and identify cytokines and cells associated with graft rejection/engraftment. 50 cytokines and 10 immune cell subsets were screened using multiplex-ELISA and flow cytometry, respectively, at baseline and PT-Days 30, 60, 100, and 180. We observed the most significant differences in cytokine levels between the engrafted and rejected groups at PT-Day 60, corresponding with clinical findings of secondary graft rejection. Of the 44 cytokines evaluated, plasma concentrations of 19 cytokines were different between the two groups at PT-Day 60. Factor analysis suggested two independent factors. The first factor (IL-17A, IL-10, IL-7, G-CSF, IL-2, MIP-1a, VEGF, and TGFb1 contributed significantly) was strongly associated with engraftment with OR = 2.7 (95%CI of 1.4 to 5.4), whereas the second factor (GROa and IL-18 contributed significantly) was not significantly associated with engraftment. Sufficient donor myeloid chimerism (DMC) is critical for the success of HSCT; here, we evaluated immune cells among high (H) DMC (DMC≥20%) and low (L) DMC (DMC<20%) groups along with engrafted and rejected groups. We found that early myeloid-derived suppressor cell (eMDSC) frequencies were elevated in engrafted patients and patients with HDMC at PT-Day 30 (P< 0.04 & P< 0.003, respectively). 9 of 20 patients were evaluated for the source of eMDSCs. The HDMC group had high mixed chimeric eMDSCs as compared to the LDMC group (P< 0.00001). We found a positive correlation between the frequencies of eMDSCs and Tregs at PT-Day 100 (r=0.72, P <0.0007); eMDSCs at BSL and Tregs at PT-Day 100 (r=0.63, P <0.004). Of 10 immune regulatory cells and 50 cytokines, we observed mixed chimeric eMDSCs and IL-17A, IL-10, IL-7, G-CSF, IL-2, MIP-1a, VEGF, TGFb1 as potential hits which could serve as prognostic markers in predicting allograft outcome towards engraftment following haploidentical HSCT employing post-transplant cyclophosphamide. The current findings need to be replicated and further explored in a larger cohort.

## 1 Introduction

Sickle cell disease (SCD) is a debilitating monogenic disorder that affects over 5 million people worldwide (1) and approximately 90,000 people in the United States (2). A substitution of valine for glutamic acid at the sixth position of the beta-globin chain in hemoglobin (Hb) leads to abnormal Hb polymerization in areas of low oxygen tension, causing recurrent vaso-occlusion. SCD is associated with early mortality and severe morbidity, including recurrent painful crises, chronic renal injury often progressing to end-stage renal disease (3, 4), avascular necrosis, stroke (5), acute chest syndrome, and cardiopulmonary complications (6). Hematopoietic stem cell transplantation (HSCT) offers a potentially curative option for SCD and can improve morbidity and overall quality of life in severely affected patients (7, 8). While human leukocyte antigen (HLA)-matched donor HSCT has high efficacy (7), this option is limited by the availability of such donors and is further complicated by the inheritance pattern of SCD (9). HLA-haploidentical (haplo) donors expand the donor pool with approximately 90% of patients having a haplo-donor (10).

Unlike hematological malignancies where complete replacement of the diseased marrow with healthy donor marrow is required, SCD does not require full donor chimerism. Using mathematical modeling, we reported that 20% donor myeloid chimerism (DMC) is sufficient to reverse SCD due to the short half-life of the sickle red blood cells (RBCs) compared to the healthy donor RBCs (11–14). We developed a non-myeloablative haplo-protocol for patients with SCD intending to maintain mixed chimerism by employing escalating doses of post-transplant cyclophosphamide (PT-Cy) (15). Graft success rate was increased with an increasing dose of PT-Cy (83% engraftment rate and 50% event-free survival rate with 100 mg/kg). The major limitation of the study, however, was the high rate of allograft rejection.

Our study therefore aimed to evaluate non-invasive prognostic cytokines and cells associated with graft rejection/engraftment in the recipients before and at defined PT time points. Understanding the transplanted patients’ immune milieu may provide cues for subsequent allograft outcome (16), either successful engraftment or allograft rejection. Here, we sought to evaluate the circulatory cytokines and immune regulatory and effector cells in peripheral blood and their intracellular cytokine-producing abilities in association with allograft outcome.

## 2 Materials And Methods

### 2.1 Patients and Samples

A total of 23 adults underwent non-myeloablative haplo-HSCT at the National Institutes of Health (NIH) from March 2010 through September 2015 for SCD (21/23) and beta-thalassemia (2/23). One patient with SCD died <6 months post-HSCT and was not included in the study. 20 patients with SCD were evaluated for cytokines and immune cell subsets (**Supplementary Table S1**). Patients were conditioned with alemtuzumab, 400 cGy total body irradiation, PT-Cy doses ranging from 0-100 mg/kg body weight in three dose dependent cohorts, (cohort 1: 0mg/kg body weight, cohort 2: 50mg/kg body weight and cohort 3: 100 mg/kg body weight). Sirolimus was loaded 1 day before transplant in cohort 1 and in the first 6 patients who received a transplant in cohort 2 and 1 day after PT-Cy in the remaining cohort 2 patients (day 4) and in all cohort 3 patients (day 5). A trough level of 10 to 15 ng/mL was targeted until 3 to 4 months posttransplant, and then the level was decreased to 10 to 12 ng/mL until 1 year posttransplant and then 5 to 10 ng/mL thereafter in engrafted patients (15). Donor engraftment was defined as sufficient donor chimerism (DMC≥20%) at PT-Day 180 and reversal of acute SCD complications. Immunophenotyping of the peripheral blood mononuclear cells (PBMCs) was performed in all available patient samples. The study was approved by the Institutional Review Board of the National Heart, Lung, and Blood Institute (NHLBI, ClinicalTrials.gov Identifier NCT00977691). All patients gave written informed consent. The study was monitored by an independent data and safety monitoring board.

Peripheral blood samples were collected at baseline (BSL) and serially at PT-Day 30, 60, 100, and 180. Blood samples were collected in EDTA tubes (Becton Dickinson, San Jose, CA, USA) and plasma stored at -80° C and PBMCs at -140° C until analysis. PBMCs were isolated using the Ficoll density gradient protocol. patients were grouped at each PT-time point based on their engraftment status [engrafted or rejected (**Supplementary Table S1**)] and DMC level [high DMC (HDMC) with ≥ 20% or low DMC (LDMC) with < 20%] (**Supplementary Table S2**).

### 2.2 Cytokine Analysis

A multiplexed magnetic bead assay was employed to analyze 48 cytokines in plasma (Bio-Rad, Hercules, CA, USA). Two cytokines [transforming growth factor-b1 (TGF-b1) and B-cell-activating factor (BAFF)] were measured using an enzyme-linked immunosorbent assay (ELISA) based DuoSet kit (R&D, Minneapolis, MN, USA). All assays were performed according to the manufacturer’s instructions. Four cytokines [interleukin (IL)-1a, IL-12p40, monocyte-chemotactic protein (MCP)-3, and tumor necrosis factor-b (TNF-b)] had more than 75% of values below the lowest limit of detection (LLOD) and two cytokines [cutaneous T-cell-attracting chemokine (CTACK), stromal cell-derived factor-1a (SDF-1a)] failed standard curves. Therefore, we excluded these cytokines from the analysis (**Supplementary Table S3**). Abbreviations for all the cytokines that are evaluated in this study are listed in **Supplementary Data**.

### 2.3 Immunophenotyping of Immune Regulatory Cells

Based on the cytokine results, two panels (**Supplementary Tables S4A, B**) were designed to evaluate various regulatory and effector immune cell subsets (**Supplementary Table S5**) by flow cytometry. Cell surface staining of PBMCs was performed as described with some modification (17). After thawing frozen vials, cells were suspended in a sterile complete medium. For surface staining, cells were stained in flow cytometry staining buffer (PBS, 2% heat-inactivated FBS), and prior to surface human antibody conjugates staining samples were treated with human FC block antibody. The immunophenotyping analysis was performed in two ways. The first analysis involved a comprehensive phenotyping of the following eight major immune cell subsets: (i) B cells: CD19^+^, (ii) CD8^+^ T cells: CD3^+^CD8^+^, (iii) regulatory T cells (Tregs): CD4^+^FoxP3^+^, (iv) effector CD4^+^ T cells: CD4^+^FoxP3^-^, (v) natural killer (NK) cells: CD3^-^CD56^+^, (vi) Monocytes: CD14^+^, (vii) dendritic cell (DC) subsets, plasmacytoid DCs (pDCs): lineage (CD3, CD19, CD56) (lin)^-^ HLA-DR^+^CD123^+^CD11c^-^ (18) and myeloid DCs (mDCs): lin^-^HLA-DR^+^CD123^-^CD11c^+^ (19), and (viii) myeloid-derived suppressor cell (MDSC) subsets (20, 21), early MDSCs (eMDSCs): lin^-^HLA-DR^-^CD11b^+^CD33^+^, monocytic MDSCs (mMDSCs): lin^-^HLA-DR^-/low^CD14^+^CD15^-^, polymorphonuclear MDSCs (PMN-MDSCs): lin^-^HLA-DR^-/low^CD14^-^CD15^+^CD11b^+^. Later more detailed analysis was performed to evaluate the following immune regulatory/effector cell types (**Supplementary Table S5**): (i) Tregs: CD4^+^CD25^+^FoxP3^+^ (22); (ii) type 1 regulatory (Tr1) cells: CD4^+^FoxP3^-^CD45RA^-^LAG3^+^CD49b^+^ (23) (iii-v) eMDSCSs, mMDSCs, and PMN-MDSCs; (vi-vii) pDCs and mDCs; (viii) regulatory B cells (Bregs): CD19^+^CD24^hi^CD38^hi^ (24) (ix) T helper (Th)1 cells: CD3^+^CD4^+^CD45RO^+^CXCR3^+^ (19), and (x) Th17 cells: CD3^+^CD4^+^CD45RO^+^CCR6^+^ (19). The gating strategies for these 10 subsets are described in **Supplementary Figures S1–S4**. The gating strategy was adapted from the referenced articles indicating each cell type and validated by the NHLBI Flow Cytometry Core. The PBMCs were first stained with cell surface markers. Then FoxP3, LAG3, TGF-b1, IL-10, and IL-7 were stained intracellularly.

#### 2.3.1 Intracellular Cytokine Staining

TGF-b1, IL-10, and IL-7 were stained intracellularly after stimulating the PBMCs with cell stimulation cocktail (phorbol 12-myristate 13-acetate, ionomycin, brefeldin A and monensin; ThermoFisher Scientific, Waltham, MA, USA) in culture medium and incubated for 5-6 hours at 37°C (25). Cells were stained with surface markers as described in the *Material and Methods* section. Then the cells were fixed using fixation and permeabilization buffer (ThermoFisher Scientific) for 30 minutes at 4°C. Fixed cells were incubated in permeabilization buffer overnight with antibodies for FoxP3 and IL-7, IL-10, and TGF-b1 cytokines at 4°C. The stained cells were acquired using multiparameter FACSymphony flow cytometer (Broomfield, CO) and analyzed by FlowJo software version10.6.2 (Tree Star, Ashland, OR, USA).

#### 2.3.2 Flow Cytometric Sample Acquisition

Samples were acquired on a Becton Dickinson Symphony flow cytometer equipped with Seven lasers (355, 407, 445, 488, 532, 633, and 785 nm wavelengths) and 35 PMT detectors, optimized as described by Perfetto et al. (26). Between 100,000 and 1x10^6^ events were collected per FCS file for each tube, depending on the number of cells available, to have sufficient events for statistical analysis of rare subsets defined by multiple markers. Data were acquired using DIVA 6.1.2 software (BD, San Jose, CA) and the analysis was performed using FlowJo™ Software (for Mac) Version 9.9.6. (Ashland, OR: Becton Dickinson and Company; 2019).

### 2.4 Statistical Methods

Mean, median, standard deviation (SD), minimum and maximum values of cytokine concentrations were calculated (**Supplementary Table S3**). LLOD categories and logistic regression model details are described in **Supplementary Data**. Additionally, we used linear regression models to compare continuous cytokine concentrations between the engrafted and rejected groups at each time point. Spearman’s rank correlations were employed to examine the correlation between the different cytokines at each time point. Factor analysis was used to examine the relationships between the selected cytokines. The factors computed based on the BSL time point for all patients were categorized into quartiles and used as predictors in logistic regression models fit to all time points for all subjects, accounting for repeated measures for the same person over time in the variance computation. Random forests using continuous cytokine levels were implemented as additional sensitivity analysis. Missing values were excluded from the analyses.

The cellular flow cytometric data highlighting the immune reconstitution were analyzed by comparing the log10-transformed frequencies. Log10-transformed frequencies were used to compare differences between the engrafted versus rejected groups and HDMC versus LDMC groups using pairwise multiple t-tests at each time point. We calculated Spearman’s rank correlations between phenotypic frequencies of immune cell subsets at each time point. All tests were two-sided, and P <0.05 was considered statistically significant. Bonferroni corrections were applied to adjust for multiple testing. Analyses were performed using STATA software (version 14.2, StataCorp LLC., College Station, TX, USA), and graphs were generated using GraphPad Prism software (version 7 and 8).

## 3 Results

### 3.1 Patient Characteristics

The characteristics of the patients in the engrafted and rejected groups and their donors are described in **Table 1A**. The engrafted group comprised of an equal number of males and females (5), whereas the rejected group consisted of 7 males and 3 females. The mean age in the engrafted group was 34.4 ± 6.8 years and in the rejected group 34.2 ± 12.21 years. More donors were female in both engrafted and rejected groups, 7/10 (70%) and 8/10 (80%), respectively. There were no significant differences between the recipient’s or donor’s age, race, gender, and cell numbers infused between the two groups (**Table 1B**).

**Table 1A:** Characteristics of the study population by transplant outcome.

Patient ID	E/R	Age	Sex	Disease	Donor Age	Donor Sex	Relation	HLA-match	CD34 **x10^6^/kg**	CD3 **x10^8^/kg**
225-03	E	37	F	HbSS	66	F	Mother	8/10	10.2	3.78
225-07	E	31	F	HbSS	60	F	Mother	7/10	13	8.07
225-19	E	36	M	HbSS	28	M	Brother	7/10	28	5.01
225-23	E	24	M	HbSS	20	M	Brother	7/10	13.4	2.59
225-33	E	37	M	HbSS	61	F	Mother	8/10	25.6	4.73
225-34	E	41	M	HbSS	45	F	Sister	8/10	15.9	5.08
225-38	E	31	F	HbSS	30	F	Sister	7/10	15.1	4.00
225-44	E	26	M	HbSS	51	F	Mother	6/10	16.8	3.95
225-43	E	47	F	HbSS	23	F	Sister	5/10	16.6	2.95
225-51	E	34	F	HbS β^0^-thal	30	M	Brother	8/10	9.70	5.28
225-10	R	36	F	HbSS	46	F	Sister	7/10	9.76	2.83
225-11	R	20	M	HbSS	47	F	Mother	6/10	15	2.65
225-16	R	47	M	HbSS	60	F	Sister	6/10	11.9	7.93
225-29	R	21	M	HbSS	51	F	Mother	5/10	12.2	3.51
225-36	R	37	M	HbSS	56	F	Mother	7/10	10.2	2.98
225-40	R	56	F	HbSC	31	M	Son	8/10	29.7	3.78
225-47	R	20	F	HbSS	51	F	Mother	6/10	10.2	6.14
225-52	R	27	M	HbSS	52	F	Mother	5/10	11.5	3.65
225-55	R	36	M	HbSS	64	M	Father	5/10	12.2	2.42
225-56	R	42	M	HbSS	23	F	Sister	7/10	10.1	6.12

E, engrafted; R, rejected; M, male; F, female; HbSS, homozygous sickle cell disease; HbSC, compound heterozygous HbS and HbC disease; HbS β^0^-thal, Compound heterozyzous HbS and β^0^ thalassemia disease.

**Table 1B:** Descriptive statistics and comparative demographics of the study population by transplant outcome.

		Engrafted N = 10 (50)	Rejected N = 10 (50)	Total N = 20 (100)	P value
**Recipient**	Age, Average years (SD) Sex, Male N (%) BMI, Average (SD) Race, N (%) o African American o Caucasian	34.4 (6.8) 5 (50.0) 23.3 (3.1) 9 (90.0) 1 (10.0)	34.2 (12.21) 7 (70.0) 23.0 (5.4) 10 (100) -	34.8 (9.6) 12 (60) 23.1 (4.5) 19 (95.0) 1 (5.0)	0.96 0.99 0.89 0.99
**Donor**	Age, Average years (SD) Sex, Male N (%) Relation, N (%) o Father o Mother o Brother o Sister o Son Gender match o Same sex, parent o Same sex, sibling o Different sex, child	41.4 (17.24) 3 (30.0) - 4 (40.0) 3 (30.0) 3 (30.0) - 2 (20.0) 4 (40.0) -	48.1 (12.55) 2 (20.0) 1 (10.0) 5 (50.0) - 3 (30.0) 1 (10.0) 2 (20.0) 1 (10.0) 1 (10.0)	44.75 (15.0) 5 (25.0) 1 (5.0) 9 (45.0) 3 (15.0) 6 (30.0) 1 (5.0) 4 (20.0) 5 (25.0) 1 (5.0)	0.33 0.99 0.99 0.99 0.99 0.99 0.500 - 0.99
**Cell number**	CD34^+^ (SD) in 10^6^ CD3^+^ (SD) in 10^8^	16.5 (6.5) 4.5 (0.9)	13.7 (5.4) 4.3 (2.1)	14.9 (5.9) 4.4 (1.6)	0.168 0.557

N, Number; SD, standard deviation; BMI, body mass index

### 3.2 Associations of Cytokine Levels With Engraftment

Among 44 cytokines evaluated, 23 with values over LLOD were further categorized into two groups: above or below the overall median of each cytokine. The remaining 21 cytokines were categorized into three groups: <LLOD, below the median, and above the median of detectable values (**Supplementary Table S3**). We first assessed the association with engraftment for all 44 cytokines (Fisher’s exact P-values given in **Table 2**). The sample at PT-Day 60 revealed the lowest P-value difference between the engrafted and rejected groups. Fibroblast growth factor (FGF), granulocyte-macrophage colony-stimulating factor (GM-CSF), IL-12p70, IL-9, and macrophage inflammatory protein (MIP)-1a were associated with engraftment (Bonferroni-corrected P <0.001), whereas granulocyte colony-stimulating factor (G-CSF), interferon (IFN)-g, IL-10, IL-13, IL-17, IL-1b, IL-1RA, IL-4, macrophage migration inhibitory factor (MIF), TGF-b1, TNF-a, and vascular endothelial growth factor (VEGF) were associated with P <0.01, and growth-regulated protein (GRO-a), platelet-derived growth factor (PDGF)-BB with P =0.05 at PT-Day 60. At PT-Day 100, IL-7 was associated with engraftment (P <0.01), as were IL-2 and VEGF (P <0.05). MIF was associated with P <0.01, and IL-7 and TGF-b1 with P <0.05 at PT-Day 180. In contrast, IL-18 was associated with rejection at PT-Day 100 with P <0.005. Notably, IL-6 was associated with engraftment at BSL (P <0.017). The remaining markers did not show any associations.

**Table 2:** Fisher’s exact test of cytokines for association with engraftment between the engrafted and rejected groups, P values (*P < 0.05, **P < 0.01, ***P < 0.001).

Cytokines	BSL	PT-Day 30	PT-Day 60	PT-Day 100	PT-Day 180
BAFF	0.350	1.000	0.620	0.650	0.622
bNGF	1.000	0.604	0.589	0.195	0.827
Eotaxin	0.170	1.000	0.153	0.650	0.622
FGF	1.000	0.303	0.000***	0.170	0.153
G-CSF	0.474	0.141	0.002**	0.370	0.050
GM-CSF	1.000	0.141	0.000***	0.170	0.335
GROa	1.000	1.000	0.024*	0.188	0.069
HGF	0.656	0.628	0.637	0.179	0.762
IFN-a2	0.656	0.170	1.000	0.484	1.000
IFN-g	1.000	0.582	0.003**	0.243	0.335
IL-10	0.211	0.170	0.002**	0.350	0.050
IL-12p70	1.000	0.139	0.001***	0.106	0.134
IL-13	0.582	0.340	0.004**	0.106	0.234
IL-15	0.408	0.232	0.718	0.777	0.485
IL-16	0.777	1.000	0.352	0.459	0.647
IL-17	1.000	0.303	0.009**	0.070	0.058
IL-18 #	1.000	0.656	0.637	0.005**	0.058
IL-1b	1.000	0.141	0.006**	0.478	0.153
IL-1RA	1.000	1.000	0.002**	0.245	0.335
IL-2	0.628	0.459	0.263	0.048*	0.350
IL-2RA	0.650	1.000	0.637	0.628	1.000
IL-3	1.000	0.187	1.000	0.714	0.377
IL-4	1.000	0.141	0.002**	0.170	0.153
IL-5	0.700	0.361	0.073	1.000	0.473
IL-6	0.017*	0.500	0.352	0.286	0.377
IL-7	1.000	0.389	0.090	0.004**	0.032*
IL-8	1.000	0.350	0.637	1.000	0.423
IL-9	1.000	0.303	0.000***	0.070	0.153
IP10	0.628	0.087	1.000	0.170	0.644
LIF	0.714	0.125	1.000	1.000	1.000
MCP-1	1.000	0.628	0.153	0.370	0.304
MCSF	1.000	1.000	0.793	0.800	0.377
MIF	1.000	0.293	0.008**	0.577	0.002**
MIG	0.350	0.087	1.000	1.000	1.000
MIP-1a	1.000	0.350	0.000***	0.170	0.050
MIP-1b	0.303	0.650	1.000	0.170	0.134
PDGF-BB	1.000	0.057	0.029*	0.070	0.058
RANTES	1.000	0.303	1.000	1.000	0.622
SCF	0.714	1.000	1.000	0.607	0.219
SCGFb	0.656	0.628	1.000	0.350	1.000
TGF-b1	1.000	0.057	0.009**	0.370	0.015*
TNF-a	1.000	0.057	0.002**	0.170	0.050
TRAIL	0.650	1.000	1.000	0.070	1.000
VEGF	1.000	0.179	0.002**	0.020*	0.335

BSL, baseline; PT, post transplantation; #, cytokine associated with rejection.

Odds ratios (ORs) from logistic models using the categories of cytokine concentrations as ordinal variables and P values for all time points are given in **Supplementary Table S6**. ORs of PDGF-BB, TGF-b1, and TNF-a were associated with engraftment with a P <0.05 at PT-Day 30. At PT-Day 60, ORs of FGF, GM-CSF, IL-9, and MIP-1a were associated with P <0.001, and G-CSF, IFN-g, IL-10, IL-17A, IL-1RA, IL-4, TGF-b1, and VEGF were associated with P <0.01; and GRO-a, IL-12p70, IL-7, MIF, and PDGF-BB with P <0.05. OR of IL-7 was associated with engraftment at PT-Day 100 with P <0.01, and IL-12p70, IL-13, IL-17, IL-2, IL-9, PDGF-BB, TNF-related apoptosis-inducing ligand (TRAIL), and VEGF were associated with P <0.05. At PT-Day 180, OR of MIF was associated with engraftment with P <0.01, whereas G-CSF, GRO-a, IL-10, IL-17A, IL-7, MIP-1a, PDGF-BB, TGF-b1, and TNF-a were associated with P <0.05. In contrast, the OR of IL-18 was associated with rejection at PT-Day 100 with P <0.01 and at PT-Day 180 with P <0.05. There were no significant differences between the two groups at BSL for any cytokines.

**Figure 1:**
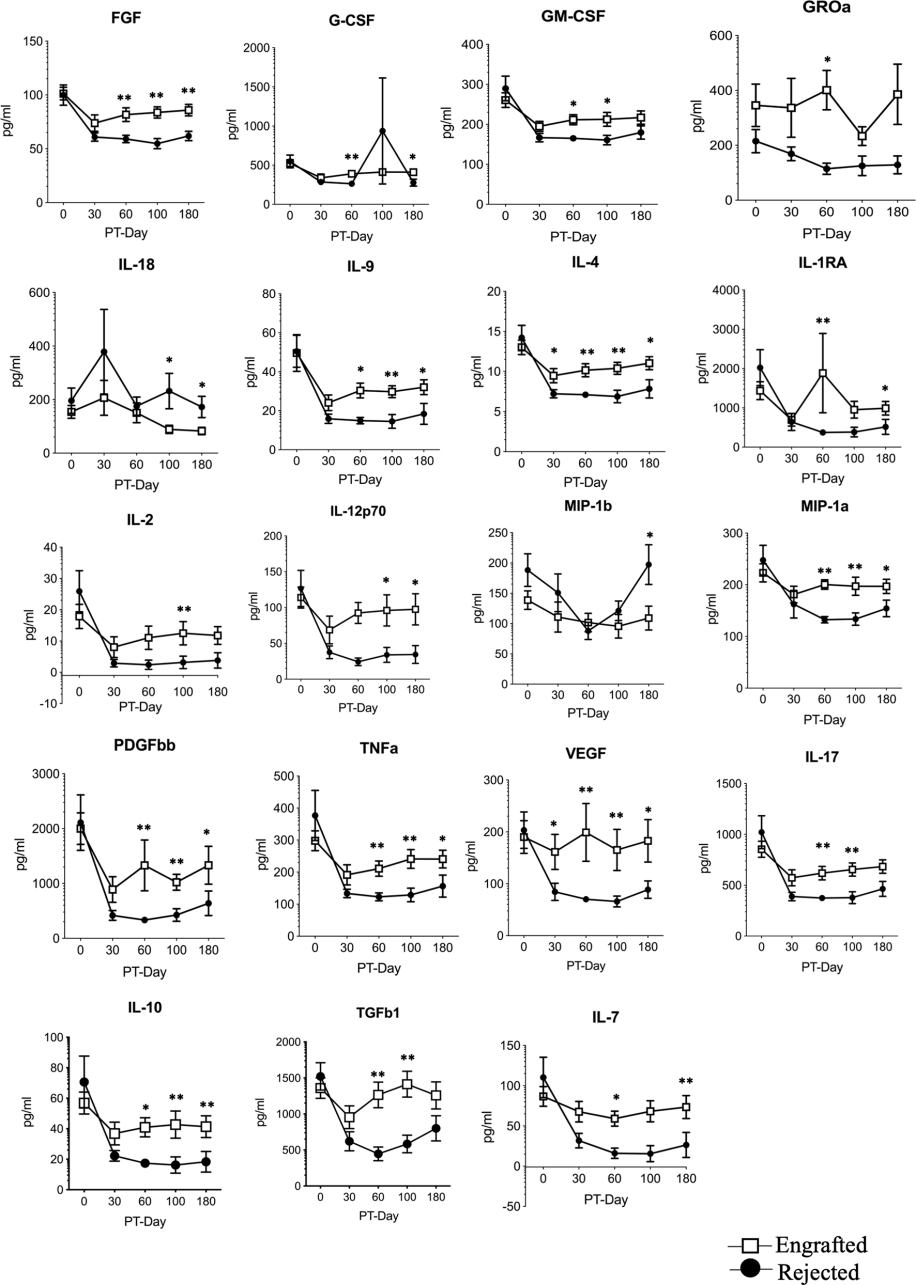
The selected differential cytokines between the engrafted and rejected groups at BSL and PT time points. Multiplex magnetic-bead based assay or ELISA for all indicated cytokines except TGF-b1was performed. Graphs shown here represent 19 differential cytokines between engrafted and rejected patients at BSL, PT-Days 30, 60, 100, and 180. Data represent the mean ± standard error, *P < 0.05, **P < 0.01.

Results from linear regression models are presented in **Supplementary Table S7**. As expected, all cytokine concentrations in plasma substantially dropped from their BSL levels after the HSCT (**Figure 1**). Further, 18 cytokine concentrations were higher in the engrafted group from PT-Day 60 to 100. Only the concentration of IL-18 was higher in the rejected group. The remaining cytokines did not show statistically significant differences in concentrations between the two groups at any time. We thus found the most significant differences in cytokine levels between the engrafted and rejected groups at PT-Day 60, the time point around which secondary graft failure typically occurs.

We used factor analysis to describe the variability among the correlated cytokines in terms of a lower number of unobserved variables called “factors” that are linear combinations of the original cytokines. After removing highly correlated cytokines, we included the following ten cytokines in a factor analysis: GROa, G-CSF, IL-10, IL-17A, IL-18, IL-2, MIP-1a, PDGF-BB, TGFb1, and VEGF. We identified two factors as important, estimated factor loadings (i.e. the coefficients in the linear combination) based on the BSL levels, and computed factors for all time points. We then categorized the factors into quartiles and used them as predictors in logistic regression models. The first factor (IL-17A, IL-10, IL-7, G-CSF, IL-2, MIP-1a, VEGF, and TGFb1 contributed significantly) was strongly associated with engraftment with OR = 2.75 (95% CI of 1.40 to 5.38) whereas the second factor (GROa, and IL-18 contributed significantly) was not statistically significant (**Supplementary Table S8**).

### 3.3 Immune Reconstitution Following Haplo-HSCT

**Figure 2:**
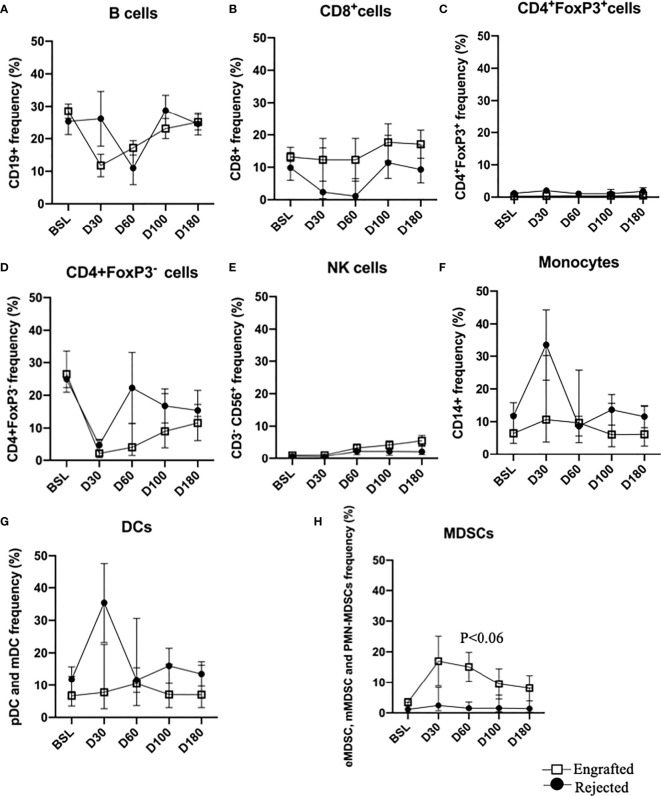
Immune reconstitution following haplo-HSCT at all time points. **(A–H)** Percent frequencies of major immune cell subsets: B cells, CD8^+^ T cells, CD4^+^FoxP3^+^ (Tregs), CD4^+^Foxp3^-^ (effector T cells), NK cells, monocytes, DCs and MDSCs at specified time points. Mean frequencies of specified immune cells are provided in the engrafted and rejected patients at BSL, PT-Days 30, 60, 100, and 180. A trend of increased MDSCs in engrafted patients is observed at PT-Day 60 (P < 0.06).

Immunophenotypic analysis of the patients’ immune cell repertoire comprising of B cells, CD8^+^ T cells, Tregs, effector CD4^+^ T cells, NK cells, monocytes, DCs (pDCs and mDCs), and MDSCs (eMDSCs, mMDSCs, and PMN-MDSCs) at BSL, PT-Days 30, 60, 100, and 180 were performed. The cellular frequencies of these cells are plotted (**Figures 2A** **–H**). A non-significant trend in the frequency of MDSCs was observed at PT-Day 60 (P >0.06). Since DMC is a critical factor in promoting allograft acceptance and treating SCD, we grouped the patients into HDMC (≥20%) and LDMC (<20%) at each time point and observed consistent PT-time point visual differences in DCs, and MDSCs between the engrafted and rejected patients between HDMC and LDMC patients (**Supplementary Figure S5 A–E**).

### 3.4 Early Myeloid-Derived Suppressor Cells Associate With Successful Graft Outcome

**Figure 3:**
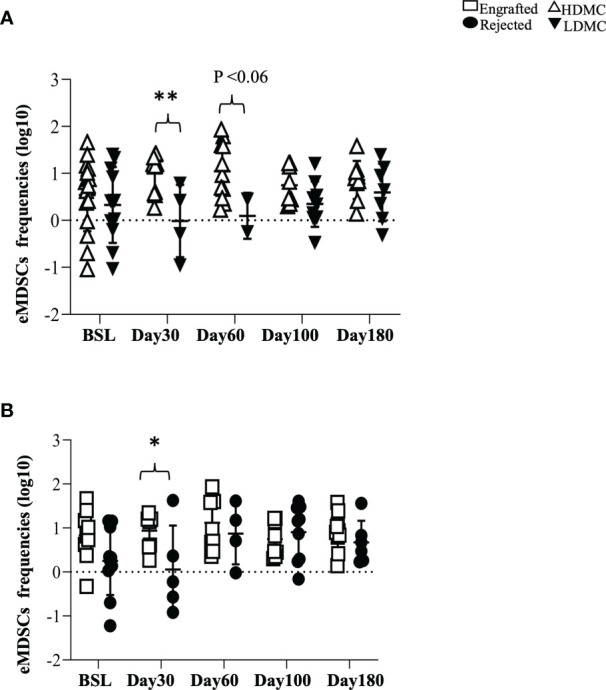
Early myeloid-derived suppressor cells associate with successful graft outcome. **(A, B)** PBMC samples were stained for eMDSCs at defined time points. **(A)** eMDSCs from HDMC and LDMC patients are plotted (PT-Day 30, ** P < 0.003) and **(B)** eMDSCs from engrafted and rejected patients are plotted (PT-Day 30, *P < 0.04).

We evaluated the percentages of three different types of MDSCs (20, 21) in our patients and compared the frequencies of each type between the engrafted and rejected groups and HDMC and LDMC groups at each time point. We observed higher frequencies of eMDSCs in the HDMC group (P <0.003; **Figure 3A** and **Supplementary Figure S6A** ) and engrafted group (P <0.04; **Figure 3B**) at PT-Day 30. We used distinguishable HLA to determine the source of eMDSCs in 9/20 patients (**Table 3**). These nine patients had a total of 16 HDMC time points and 14 LDMC time points. The source of eMDSCs revealed the following patterns: in engrafted patient 225-19 up to 99% eMDSCs were donor-derived at all time points, wherein with rejected patients 225-10, 225-52, and 225-55, all eMDSCs were 100% recipient-derived in origin. Interestingly, in rejected patient 225-40, at day 30-PT, eMDSCs were 100% recipient-derived. At later time points, however, eMDSCs were 100% from the donor. Other patients maintained more mixed donor and recipient origins until at least day 180-PT. eMDSCs from both donor and recipient (mixed chimeric state) origins were observed at 15/16 HDMC time points as compared to only two LDMC time points (**Table 3**; P< 0.00001 and **Supplementary Figures S6B, C**).

**Table 3:** Source of early myeloid-derived suppressor cells at specified post-transplantation time points.

Patient ID	E/R	HDMC/LDMC	Distinguishable HLA	PT time point	eMDSCs Recipient (%)	eMDSCs Donor (%)	Mixed Chimerism present
225-19	E	HDMC	Recipient is A3+	PT-Day30	1	99	Yes
		HDMC		PT-Day60	1	99	Yes
		HDMC		PT-Day180	1	99	Yes
225-43	E	HDMC	Donor is A2+	PT-Day30	76	24	Yes
		HDMC		PT-Day100	85	15	Yes
		HDMC		PT-Day180	82	18	Yes
225-51	E	HDMC	Donor is A2+	PT-Day60	96	4	Yes
		HDMC		PT-Day100	98	2	Yes
		HDMC		PT-Day180	94	6	Yes
225-44	E	HDMC	Donor is A2+	PT-Day30	50	50	Yes
		HDMC		PT-Day60	99	1	Yes
		HDMC		PT-Day100	91	9	Yes
		HDMC		PT-Day180	84	16	Yes
225-10	R	LDMC	Donor is A3+	PT-Day30	100	0	No
		LDMC		PT-Day60	100	0	No
		LDMC		PT-Day100	100	0	No
225-52	R	LDMC	Donor is A3+	PT-Day30	100	0	No
		LDMC		PT-Day60	100	0	No
		LDMC		PT-Day100	100	0	No
		LDMC		PT-Day180	100	0	No
225-55	R	LDMC	Donor is A2+	PT-Day60	100	0	No
		LDMC		PT-Day100	100	0	No
		LDMC		PT-Day180	100	0	No
225-36	R	HDMC	Donor is A2+	PT-Day30	56	44	Yes
		HDMC		PT-Day60	98	2	Yes
		LDMC		PT-Day100	99	1	Yes
		LDMC		PT-Day180	99	1	Yes
225-40	R	HDMC	Donor is A2+	PT-Day30	0	100	No
		LDMC		PT-Day100	100	0	No
		LDMC		PT-Day180	100	0	No

Table showing the source of eMDSCs at HDMC and LDMC time points. High chimerism in eMDSCs observed in HDMC as compared to LDMC groups (chi-square =17.099; P< 0.00001, Yates correction applied).

E, engrafted; R, Rejected; HDMC, high donor myeloid chimerism; LDMC, low donor myeloid chimerism; PT, post-transplant; eMDSCs, early myeloid-derived suppressor cells.

### 3.5 Evidence of High Frequencies of Tregs in Engrafted and HDMC Patients

**Figure 4:**
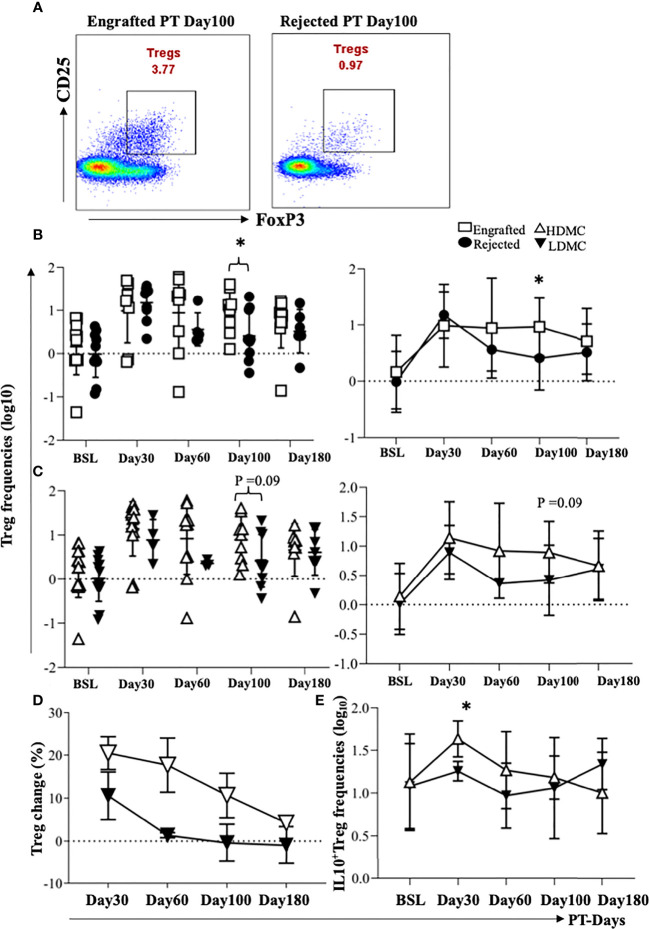
Evidence of high frequencies of Tregs in engrafted and HDMC patients. **(A)** PBMC samples were stained for Tregs, and representative plots of Tregs from an engrafted and rejected patient at PT Day 100 are shown. **(B)** Individual and mean Treg frequencies from engrafted and rejected groups are plotted (PT-Day 100, *P < 0.04). **(C)** Individual and mean Treg frequencies from each sample in HDMC and LDMC groups are plotted. **(D)** Percent change in the frequencies of Tregs at different time PT with respect to BSL was plotted in HDMC and LDMC groups. **(E)** Tregs were intracellularly stained for IL-10 and mean IL-10 producing Tregs were plotted within HDMC and LDMC groups (PT-Day 30, *P < 0.02).

Tregs are the most commonly observed cellular population in patients with immune tolerance (27), and they prevent acute graft versus host disease (GVHD) (28) following HSCT. We compared frequencies of Tregs between the engrafted and rejected patients and among HDMC and LDMC groups. While we did not find any significant differences after multiple testing correction, we noticed a trend towards increased frequencies of Tregs in the engrafted group at PT-Day 100, (P <0.04; **Figures 4A, B**) and in the HDMC group at PT-Day 100 (P <0.09; **Figure 4C**). The elevated frequencies of Tregs agree with our cytokine results, where we observed elevated plasma levels of IL-10 at PT-Day 60 (P <0.05), PT-Day 100 (P <0.01), and PT-Day 180 (P <0.05) in engrafted patients. We tracked the source of Tregs using distinguishable HLA in 9/20 patients. We did not observe statistically significant differences between mixed chimeric or non-chimeric Tregs in HDMC and LDMC groups (**Table 4**).

**Table 4:** Source of Tregs at specified post-transplantation time points.

Patient ID	E/R	HDMC/LDMC	Distinguishable HLA	PT time point	TregsRecipient (%)	TregsDonor(%)	Mixed Chimerism present
225-19	E	HDMC	Recipient is A3+	PT-Day30	100	0	No
		HDMC		PT-Day60	100	0	No
		HDMC		PT-Day180	100	0	No
225-43	E	HDMC	Donor is A2+	PT-Day30	99	1	Yes
		HDMC		PT-Day60	99	1	Yes
		HDMC		PT-Day180	98	2	Yes
225-51	E	HDMC	Donor is A2+	PT-Day60	100	0	No
		HDMC		PT-Day100	100	0	No
		HDMC		PT-Day180	100	0	No
225-44	E	HDMC	Donor is A2+	PT-Day30	93	7	Yes
		HDMC		PT-Day60	99	1	Yes
		HDMC		PT-Day100	100	0	No
		HDMC		PT-Day180	96	4	Yes
225-10	R	LDMC	Donor is A3+	PT-Day30	100	0	No
		LDMC		PT-Day60	100	0	No
		LDMC		PT-Day100	100	0	No
225-52	R	LDMC	Donor is A3+	PT-Day30	100	0	No
		LDMC		PT-Day60	100	0	No
		LDMC		PT-Day100	100	0	No
		LDMC		PT-Day180	100	0	No
225-55	R	LDMC	Donor is A2+	PT-Day60	100	0	No
		LDMC		PT-Day100	100	0	No
		LDMC		PT-Day180	100	0	No
225-36	R	HDMC	Donor is A2+	PT-Day30	100	0	No
		HDMC		PT-Day60	100	0	No
		LDMC		PT-Day100	100	0	No
		LDMC		PT-Day180	100	0	No
225-40	R	HDMC	Donor is A2+	PT-Day30	100	0	No
		LDMC		PT-Day60	100	0	No
		LDMC		PT-Day100	100	0	No
		LDMC		PT-Day100	95	5	Yes

Table showing the presence or absence of mixed chimerism in Tregs at HDMC and LDMC time points (chi-square =2.63; P< 0.10, Yates correction applied). No differences were observed between the two groups.

E, engrafted; R, Rejected; HDMC, high donor myeloid chimerism; LDMC, low donor myeloid chimerism; PT, post-transplant.

We further calculated the percent change in the frequencies of Tregs from the BSL for each patient and compared the percent change between HDMC versus LDMC groups. We observed a higher Treg change in HDMC patients (**Figure 4D**). Although we observed no significant correlation between the frequencies of Tregs and percentages of DMC, frequencies of Tregs mirrored the DMC dynamics. This was observed in two of the patients who engrafted initially before they rejected their grafts at PT-Days 60 and 100 respectively. The frequencies of Tregs at these time points decreased close to BSL as opposed to one patient who maintained engraftment and high frequency of Tregs persisted (**Supplementary Figure S7A–C**). Further, we evaluated IL-10 and TGF-b1 producing Tregs and found a trend of higher IL-10 producing Tregs in the HDMC group at PT-Day 30 (P <0.02; **Figure 4E**), however, TGF-b1 producing Tregs did not show any difference between groups (data are not shown).

Plasma cytokine data revealed higher levels of IL-17 in the engrafted patients at PT-Days 60 and 100 (**Figure 1**). However, we did not observe any statistically significant difference in the frequencies of Th17 cells between engrafted and rejected patients and HDMC and LDMC groups (data are not shown). We also did not find a statistically significant difference in TGF-b1 and IL-10 producing Th17 cells between the HDMC and LDMC groups nor the frequencies of Bregs, pDCs, mDCs, mMDSCs, PMN-MDSCs, and Tr1 cells either between the engrafted and rejected or the HDMC and LDMC groups (data not shown).

### 3.6 Early Myeloid-Derived Suppressor Cells and Tregs Correlate Positively With Each Other

**Figure 5:**
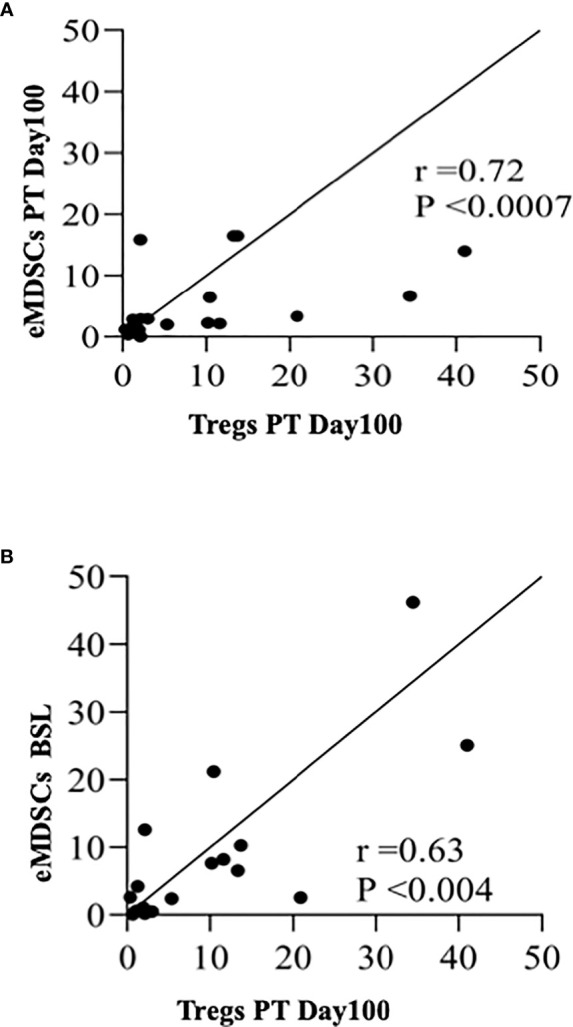
Early myeloid derived suppressor cells and Tregs correlate positively with each other. **(A)** Spearman rank correlation between Tregs at PT-Day100 and eMDSCs at PT-Day 100 (r=0.72; P < 0.0007). **(B)** Spearman rank correlation between Tregs at PT-Day 100 and eMDSCs at BSL (r = 0.63; P < 0.004) are shown.

Since Tregs have been associated with tolerance, we next performed the correlation analysis between eMDSCs and Tregs and observed a positive correlation between the frequencies of eMDSCs and Tregs at PT-Day 100 (r=0.72, P <0.0007; **Figure 5A**). Importantly, Tregs at PT-Day 100 correlated positively with the eMDSCs at BSL (r=0.63, P <0.004; **Figure 5B**). Tregs at PT-Day 60 tend to show positive correlation with eMDSCs at PT-Day 180 but the association was not significant after applying correction for multiple testing (**Supplementary Figure S8A**). We next tested the correlation of the frequencies of eMDSCs with percentages of DMC at all PT time points. We observed a trend towards a positive correlation between frequencies of eMDSCs at PT-Day 60 with the percentage of DMC at PT-Day 180, but the association could not stand the correction applied for multiple testing (**Supplementary Figure S8B**).

We next evaluated the number of patients at each post-transplantation time point who experienced graft failure with donor myeloid chimerism (DMC) levels below 20% as an indicator of graft failure based on Kaplan Meier estimates (**Supplementary Table S9**). Of the total 20 patients, DMC levels decreased below 20% at PT-Day 30 in 3 patients. At PT-Day 60, 4 additional patients had their DMC below 20% and at PT-Day 100, 5 additional patients. Finally, DMC levels decreased below 20% in 6 additional patients at PT-Day 180, adding up to 11 patients with graft failure in total. The time to graft failure (DMC below 20%) is also plotted in **Supplementary Figure S9**, that also shows numbers of subjects at risk for graft failure at each time point.

## 4 Discussion

Allograft rejection is a complex process involving an interplay between different cells and multiple cellular mediators. Although several promising molecular targets for early detection of GVHD and response to its treatment are known (29, 30), reliable biological markers to identify graft rejection in HSCT still do not exist (31, 32). In this study, we evaluated the plasma levels of 44 cytokines and 10 immune regulatory and effector cells with an aim to get target cell populations and cytokines for our future studies. Since adequate donor myeloid chimerism is critical and predictive of positive allograft status in terms of resolution of SCD related symptoms (15, 33),we evaluated the cellular data between patients with high (≥20%) and low (<20%) donor myeloid chimerism levels.

Since HSCT conditioning regimens usually lead to the potent induction and release of pro-inflammatory cytokines as a reflection of severe systemic inflammation (34), we identified several pro-inflammatory and regulatory cytokines, chemokines, and growth factors that were associated with successful engraftment at PT-Day 60. We observed increased expression of G-CSF, GM-CSF, IFN-g, IL-1b, IL-2, IL-4, IL-7, IL-10, IL-12p70, IL-17A, MIP-1a, TNF-a, TGF-b1, and VEGF in successfully engrafted patients. These cytokines reflect hematopoiesis of engrafted cells (35), activation of T (36), B (37), and macrophage differentiation (38) and induction of tolerance (39). Notably, we identified only one marker, IL-18, which was downregulated in engrafted patients and stayed at a low level through PT-Day 180. An important role of IL-18 in allograft rejection has been postulated in a recent study using a rat model of liver transplantation, which showed that specific suppression of IL-18 was associated with significantly decreased serum alanine aminotransferase levels, diminished histologic hepatic injury early after transplantation, and prolonged allograft survival (40).

MDSCs have gained attention for their potential role in allograft tolerance following heart and islet transplantation in mice (41, 42) along with renal transplantation in rats (43). The pro-inflammatory environment, which induces the development of MDSCs in cancer and infection, mimics the anti-donor response following transplantation (44–47). Our data revealed an increased and consistent presence of MDSCs in engrafted patients starting at PT-Day 30 and onwards. Notably, we observed elevated G-CSF, GM-CSF, IL2, VEGF, IL-1b, FGF, TNF-a, TGF-b1, and IL-10 levels, which are reported to be the drivers of MDSC activation (48, 49), sustenance (50), and suppressive activity (51, 52). MDSC subpopulations are hypothesized to be highly plastic, and little is known about their relevance in transplantation. A renal transplantation study in humans revealed mMDSCs to be present in the peripheral blood of tolerant patients (53). A recent study demonstrated the significance of eMDSCs in controlling acute GVHD following allo-HSCT in humanized mice (54). Here, we observed that the frequencies of eMDSCs are elevated at early time point PT-Day 30 in HDMC patients.

MDSCs favor mixed chimerism in a combined murine bone marrow-cardiac transplantation model and control anti-donor T cell response *in vitro* (55). The DMC level at PT-Day 180 correlated positively with the frequencies of eMDSCs at PT-Day 60 (r=0.45, P <0.04), which suggests they have a role in maintaining high levels of DMC. More than 90% (93.8%) of chimeric eMDSCs in the HDMC group compared to less than 10% (6.6%) in the LDMC group bolsters the relevance of the promotion of chimerism in promoting graft acceptance. We observed that the presence of donor MDSCs promoted allograft acceptance as all the engrafted patients and all HDMC timepoints had them. Although it could be presumed that the high chimerism status at these time points account for their donor derived origin but a recent murine study revealed that donor MDSCs promote cardiac allograft tolerance *via* induction of recipient derived MDSCs (56).

MDSCs suppressive activity is based on their ability to directly suppress proliferation of effector T, B, and NK cells by expressing inducible nitric oxide synthase and arginase (57) and by modifying IFN-g and IL­10 dependent T cell differentiation pathways, which promote Treg differentiation (58). Ample evidence indicates robust crosstalk between MDSCs and Tregs favoring immunosuppression (59–61). Indeed, we observed a positive correlation between eMDSCs at PT-Day 100 and Tregs at PT-Day 100 (r=0.72, P <0.0007). Notably, a positive correlation was also observed between eMDSCs at BSL and Tregs at PT-Day100 (r=0.63, P <0.004), which suggests a possible synergistic association between eMDSCs at BSL in promoting graft tolerance by increasing Tregs.

We observed evidence of increased Tregs at PT-Day 100 in engrafted patients. However, the association was not significant after Bonferroni correction was applied for correction of multiple testing, possibly due to limited sample size which further is reduced when comparisons are made at specific PT time points. We also observed the change in the frequencies of Tregs in the HDMC group from BSL following HSCT was higher than the LDMC group. Interestingly, with seven time points where chimeric Tregs were observed, six belonged to the HDMC time point group and only 1 to the LDMC group, which supports chimerism favoring tolerance. Further, Tregs showed a trend towards increased IL-10 at PT-Day 30 in the HDMC group, suggesting their active presence in allograft acceptance. Tregs comprise the major arm of immunosuppression (22) and their presence in the engrafted patients is therefore not surprising. Tregs mediate their suppressive function through a variety of different mechanisms (62, 63) including the production of the anti-inflammatory cytokine, IL-10 (64). A significant elevation of IL-10 in engrafted patients at various times post-HSCT validates their immune-suppressive activity and functionality. IL-10 serves to directly or indirectly inhibit effector T-cell responses by inhibiting cytokine production, suppressing Th1 and Th2 cell proliferation, and downregulating major histocompatibility complex class II on monocytes (65–70). We observed statistically significantly elevated plasma levels of IL-17 in engrafted patients at PT-Day 60 and 100. However, the frequencies of Th17 cells were not significantly different between engrafted versus rejected patients and HDMC versus LDMC groups. Growing evidence suggests that Tregs are highly plastic with the potential to convert into pro-inflammatory Th17 cells (71, 72). However, we did not examine the plasticity of either Treg or Th subsets in our study.

Based on our observations, the allograft outcome may be determined by the complex molecular network of pro-inflammatory and anti-inflammatory cytokines along with the relative presence of effector and suppressive cells. In accordance, we observed that the increased presence of mixed chimeric eMDSCs and Tregs could be associated with tolerance in our study.

There were several limitations to this study. First, the small sample size limited statistical power and the data came from a single institution. Because samples at all PT-time points were limited and only as early as day 30 PT, we do not know whether our findings represent a pre-rejection trend or a post-rejection phenomenon. More frequent sampling, especially at early time points, may also help to assess real-time characterization of immunological tolerance. We could not evaluate the source of eMDSCs and Tregs in all the samples due to unavailability of distinguishable HLA antibodies. Further, due to limited cells, we were unable to perform *in vitro* suppression studies to evaluate whether eMDSCs and Tregs from engrafted patients could mediate better immune suppression of effector T cells. Although we evaluated the data concerning the three sub cohorts, no cyclophosphamide, low dose cyclophosphamide, and high dose cyclophosphamide, and observed no statistical differences in the frequency of various cellular fractions, the numbers were also too small for sufficient statistical power. We did not evaluate the variability that might have occurred due to variations in sirolimus dosages. In addition, post-transplant CMV reactivation or other common infections could be confounding factors too which could not be adequately assessed due to the small sample size.

In summary, among the evaluated cells, mixed chimeric eMDSCs were present differentially in the two groups with varied outcomes. Our future trials will also focus on evaluating the presence of eMDSCs and their origin early after transplantation and their *in-vitro* suppressive abilities. In addition, Tregs and IL-10 producing Tregs showed higher trends in the HDMC group which will be evaluated in a subsequent cohort. Here, our data demonstrate that the presence of mixed chimeric eMDSCs at early time points, elevated plasma levels of IL-10 and TGF-b1, and IL-10 producing Tregs could serve as potential prognostic markers in predicting the allograft outcome following haploidentical HSCT employing similar pre and post-transplant conditioning for SCD. eMDSCs and the associated cytokines (G-CSF, GM-CSF, IL2, VEGF, IL-1b, FGF, TNF-a, TGF-b1, IL-10), and the rest of first-factor plasma cytokines (IL-17A, IL-7, MIP-1a) will be further validated in a larger cohort with frequent post-transplant time points.

## Data Availability Statement

The original contributions presented in the study are included in the article/**Supplementary Material**. Further inquiries can be directed to the corresponding author.

## Ethics Statement

The study was approved by the Institutional Review Board of the National Heart, Lung, and Blood Institute (NHLBI, ClinicalTrials.gov Identifier NCT00977691). The patients/participants provided their written informed consent to participate in this study.

## Author Contributions

DB designed, performed, and analyzed the flow cytometry data and wrote the manuscript. PO designed, performed, and analyzed the multiplex experiments and wrote the manuscript. AG designed and performed flow experiments and reviewed the manuscript. PD assisted with flow cytometry panel designing and calibration and reviewed the manuscript. AS assisted with flow cytometry experiments and reviewed the manuscript. RP helped with statistical analysis and reviewed the manuscript. JM helped to analyze flow cytometry data and reviewed the manuscript. FS and MP helped with cytokine data analysis and reviewed the manuscript. CP, AB, GF, and BF participated in performing the experiments and reviewed the manuscript. CF conceived the study, designed, and analyzed experiments and reviewed the manuscript. All authors contributed to the article and approved the submitted version.

## Conflict of Interest

The authors declare that the research was conducted in the absence of any commercial or financial relationships that could be construed as a potential conflict of interest.

## Publisher’s Note

All claims expressed in this article are solely those of the authors and do not necessarily represent those of their affiliated organizations, or those of the publisher, the editors and the reviewers. Any product that may be evaluated in this article, or claim that may be made by its manufacturer, is not guaranteed or endorsed by the publisher.
