# Supplementary material for: Early Myeloid Derived Suppressor Cells (eMDSCs) Are Associated With High Donor Myeloid Chimerism Following Haploidentical HSCT for Sickle Cell Disease
Source: Front Immunol. 2021 Nov 30;12:757279. doi: 10.3389/fimmu.2021.757279 (PMC8669726; doi:10.3389/fimmu.2021.757279)
Supplement: Supplementary file 1 [file DataSheet_1.pdf]

Supplementary data

Abbreviations:

BAFF	B-cell-activating factor
Bregs	regulatory B cells
CIs	confidence intervals
CTACK	cutaneous T-cell-attracting chemokine
ELISA	enzyme-linked immunosorbent assay
FGF	fibroblast growth factor
G-CSF	granulocyte colony-stimulating factor
GM-CSF	granulocyte-macrophage colony-stimulating factor
GRO	growth-regulated protein
GVHD	graft versus host disease
HLA	human leukocyte antigen
HSCT	hematopoietic stem cell transplantation
IFN	interferon
IL	interleukin
IP	interferon gamma-induced protein
LLOD	lowest limit of detection
MCP	monocyte-chemotactic protein
MDSCs	myeloid derived suppressive cells
MIF	macrophage migration inhibitory factor
MIP	macrophage inflammatory protein
MMP	matrix metallopeptidase
NK	natural killer
ORs	odds ratios
PDGF	platelet-derived growth factor
PT	post-transplant
SCD	sickle cell disease
SDF	stromal cell-derived factor
TGF	transforming growth factor
TNF	tumor necrosis factor
TRAIL	tumor necrosis factor-related apoptosis-inducing ligand
Tregs	regulatory T cells
Tr1 cells	T regulatory type 1 cells
VEGF	vascular endothelial growth factor

Supplementary Methods

LLOD categories and logistic regression model

We generated three ordered categories of cytokine concentrations: values below LLOD were one category and those above LLOD were categorized into two groups based on above and below the median of values above LLOD. Fisher's exact test was used to compare categorical cytokine levels between the engrafted and rejected groups. Odds ratios (ORs), 95% confidence intervals (CIs), and *p* trend values were calculated by treating the categories of cytokine concentrations as ordinal variables in logistic regression models to fit the engrafted and rejected groups.

Supplementary figure legends

Figure S1. Gating strategies for Tregs and Tr1 cells. PBMCs were stained for Tregs and Tr1 cells. Flow cytometry gating strategies are shown for $CD4^+CD25^+FoxP3^+$ Treg population and $CD4^+CD45RA^-FoxP3^-LAG3^+CD49b^+$ Tr1 cell population.

Figure S2. Gating strategies for eMDSCs and Bregs. PBMCs were stained for different subsets of MDSCs. Flow cytometry gating strategies are shown for $Lin^-CD3^-HLA\text{-}DR^-$ $CD11b^+CD33^+$ eMDSC population and for $CD19^+CD24^{hi}CD38^{hi}$ Breg population.

Figure S3. Gating strategies for mMDSCs, PMN-MDSCs, pDCs and mDCs. PBMCs were stained for different subsets of MDSCs, pDCs, mDCs. Flow cytometry gating strategies are shown for $Lin^-HA\text{-}DR^{-/low}CD14^+CD15^+$ mMDSC population, and $Lin^-HA\text{-}DR^{-/low}CD14^-$ 15^+CD11b^+ PMN-MDSC population. pDC population gated as $CD19^-CD3^-CD56^-HLA\text{-}$ $DR^+CD11c^-CD123^+$, and mDC population as $CD19^-CD3^-CD56^-HLA\text{-}DR^+CD123^-$ $CD11c^+$.

Figure S4. Gating strategies for Th1 and Th17 cells. PBMCs were stained for Th1 and Th17 cell subsets. Flow cytometry gating strategies are shown for $CD3^+CD4^+CD45RO^+CXCR3^+$ Th1 cells and $CD3^+CD4^+CD45RO^+CCR6^+$ Th17 cells.

Figure S5. Heatmap showing the log10 cellular frequencies of major immune cell subsets: B cells, $CD8^+$ T cells, $CD4^+FoxP3^+$ (Tregs), $CD4^+Foxp3^-$ T cells, NK cells, macrophages, DCs and MDSCs distribution at defined time points (A) BSL, and **(B-E)** PT-Days 30, 60, 100, and 180 in HDMC and LDMC patients.

Figure S6. Early myeloid-derived suppressor cells associate with successful graft outcome
(A) Representative plots of HDMC patient at PT-Day 30 and LDMC patient at the same time point are shown. **(B)** Representative plots of HDMC patient at PT-Day 30 showing the presence of eMDSCs both from recipient (75.6%) and donor (22.6%) origin. Donor is HLA $A2^+$ and recipient is HLA $A2^-$. **(C)** Representative plots of LDMC patient at PT-Day 100 showing the absence of chimeric eMDSCs. Only recipient-derived eMDSCs could be seen (99.9%). Donor is HLA $A2^+$ and recipient is HLA $A2^-$.

Figure S7. Tregs showed direct proportionality with recipient DMC levels. PBMCs from 3 different patients were stained and analyzed for Tregs. **(A-B)** Kinetics of Tregs with DMC from two patients who showed early engraftment and then rejection at PT-Day 60 and PT-Day 100 and **(C)** a patient who showed engraftment until the end of the study, PT-Day 180.

Figure S8. Early myeloid derived suppressor cells and Tregs correlate positively with each other

(A) Spearman rank correlations between Tregs at PT-Day 60 and eMDSCs at PT-Day 180 ($r=0.59$; * $P<0.03$). **(B)** Spearman rank correlations between eMDSCs at PT-Day 60 and DMC at PT-Day 180 ($r=0.45$; * $P<0.04$) are plotted.

Figure S9. Kaplan Meier estimates based on donor myeloid chimerism at each time point. Kaplan Meier plot shows when donor myeloid chimerism levels are at risk for decreasing below 20% in individuals.

Supplementary Tables

Table S1. Plasma samples and peripheral blood mononuclear cells collected from study subjects by patient ID over time post-transplantation

	Patient ID	BSL Plasma	BSL PBMNC	PTD 30 Plasma	PTD 30 PBMNC	PTD 60 plasma	PTD 60 PBMNC	PTD 100 plasma	PTD 100 PBMNC	PTD 180 Plasma	PTD 180 PBMNC
E	225-03	X	X	X	X	X	X	X	X	X	X
	225-07	X	X	X	X	X	X	X	X	X	X
	225-19	X	X	X	X	X	X	X	–	X	X
	225-23	X	X	X	X	X	X	X	X	X	X
	225-33	X	X	X	-	X	X	X	X	X	X
	225-34	X	X	X	X	X	X	–	-	X	X
	225-38	X	X	X	X	X	X	X	X	X	X
	225-44	X	X	X	X	X	X	X	X	X	X
	225-43	X	X	X	X	–	–	X	X	X	X
	225-51	X	X	–	–	X	X	X	X	X	X
R	225-10	X	X	X	X	X	X	X	X	X	–
	225-11	X	X	X	X	X	-	X	X	–	-
	225-16	X	X	X	X	X	X	X	X	–	X
	225-29	X	X	X	X	X	-	X	X	X	X
	225-36	X	X	X	X	X	X	X	X	X	X
	225-40	X	X	X	X	X	X	X	X	X	X
	225-47	X	X	X	X	X	-	X	X	X	-
	225-52	X	X	X	X	X	X	X	X	X	X
	225-55	X	X	X	-	-	-	X	X	X	X
	225-56	X	X	X	X	–	-	X	X	–	-

E, engrafted; R, rejected; BSL, baseline; PTD, post-transplant day; PBMNC, peripheral blood mononuclear cells; X, available samples.

Table S2. Categorization of the patients by DMC percentages (HDMC or LDMC)

Group	Patient ID	BSL	DMC PT-Day 30	DMC PT-Day 60	DMC PT-Day 100	DMC PT-Day 180
**HDMC**	225-19	X	100	100		94
	225-44	X	98	99	97	97
	225-43	X	99		92	98
	225-51	X		83	50	44
	225-33	X		96	79	78
	225-03	X	100	99	94	78
	225-34	X	88	100		100
	225-38	X	100	98	95	92
	225-36	X	98	62		
	225-40	X	99			
	225-47	X	100			
	225-16	X	100	99	98	
	225-07	X	100	66	26	
	225-23	X	99	87	63	34
	225-56	X	64			
**LDMC**	225-55	X		0	0	0
	225-10	X	0	0	0	
	225-52	X	0	0	0	0
	225-11	X	0		0	
	225-29	X	6		0	0
	225-36	X			0	0
	225-40	X		0	0	0
	225-47	X			0	
	225-16	X				0
	225-56	X			8	
	225-07	X				18

BSL, baseline; PT, post transplantation; DMC, donor myeloid chimerism; HDMC, high donor myeloid chimerism; LDMC, low donor myeloid chimerism.

Table S3. Descriptive statistics and categories used in Fisher's exact test of the 44 evaluated cytokines in plasma samples

Cytokines	Mean	SD	Min	Max	Median	Number of samples		
						LLOD	below median	above median
BAFF	3,868	2,219	112	8,889	3,568		46	46
bNGF	3.48	2.24	0.13	10.23	2.77	31	30	31
Eotaxin	96.11	37.34	47.78	257.95	87.04		46	46
FGF	77.62	26.29	33.70	175.66	73.97		46	46
G-CSF	449.6	713.2	133.6	7,034.4	346.8		46	46
GM-CSF	207.5	64.6	107.9	490.1	192.4		46	46
GROa	259.6	211.5	24.3	1,050.7	180.1	4	44	44
HGF	581.1	799.0	104.6	6,301.5	337.3	2	45	45
IFN-a2	59.87	21.69	5.12	101.75	58.01	2	45	45
IFN-g	859.0	504.4	273.7	3,095.4	762.3	6	43	43
IL-10	37.23	29.99	1.65	173.42	29.46		47	45
IL-12p70	80.52	59.30	7.31	259.16	64.64	7	43	42
IL-13	168.4	85.0	47.2	497.2	150.1	6	44	42
IL-15	25.57	29.14	1.89	151.33	17.22	60	16	16
IL-16	614.8	418.1	129.4	2,360.2	506.3	13	39	40
IL-17A	610.4	316.8	196.8	2,024.1	548.2		46	46
IL-18	186.6	212.7	31.3	1,548.0	126.3		46	46
IL-1b	60.61	38.71	5.58	237.86	56.98	2	45	45
IL-1RA	1,036	1,255	76	9,914	735	2	45	45
IL-2	15.49	12.76	0.59	62.00	11.36	31	31	30
IL-2RA	114.7	67.4	8.2	340.3	106.6		47	45
IL-3	241.2	141.2	36.5	565.4	224.0	37	27	28
IL-4	9.84	3.62	3.48	23.32	9.25		47	45
IL-5	18.28	13.02	1.94	83.31	15.24	28	32	32
IL-6	17.21	9.97	0.84	60.56	15.05	18	37	37
IL-7	71.94	46.45	5.93	288.93	66.75	19	37	36
IL-8	25.67	18.43	2.65	89.24	21.07	2	45	45
IL-9	28.63	20.07	5.84	122.75	24.61		46	46
IP10	1,558	1,223	184	5,774	1,272		46	46
LIF	14.24	11.53	0.41	61.21	10.63	18	37	37
MCP-1	238.1	111.9	83.2	680.8	219.8		46	46
MCSF	10.40	9.61	0.37	53.38	7.37	7	42	43
MIF	474.0	286.7	88.5	1,848.8	390.3	11	40	41
MIG	1,819	2,915	115	20,444	970		46	46
MIP-1a	184.7	64.4	88.4	436.2	167.4		46	46
MIP-1b	129.8	73.3	15.1	367.6	114.4		46	46
PDGF-BB	1,078	1,057	66	5,706	746		46	46
RANTES	9,863	2,423	5,766	21,253	9,604		46	46
SCF	50.96	48.07	2.48	217.61	34.00	17	37	38
SCGFb	32,658	17,505	9,068	98,630	26,920		46	46
TGF-b1	1,036	593	89	2,224	1,012		46	46
TNF-a	213.6	131.6	67.4	908.0	186.8		46	46
TRAIL	58.75	32.59	0.94	167.82	53.11		47	45
VEGF	143.3	109.3	23.7	610.5	112.1		46	46

LLOD, lowest limit of detection; SD, Standard deviation; for cytokines-refer to abbreviations above

Table S4 Flow cytometry panels to evaluate the immune cell subsets
A)

Cell Markers	Fluorochrome	Vendor	Catalog no.	Clone
CD33	BV421	BD	562854	WM53
Live Dead	Aqua Yellow	Invitrogen	L34968	-
CD11c	BV605	Biolegend	301635	3.9
CD24	BV650	BD	563720	ML5
HLA-DR	BV786	BD	564041	G46-6
CD38	PE-Cy5	BD	555461	HIT2
CD3<br>CD56	PerCP-Cy5.5<br>PerCP-Cy5.5	eBioscience	46003641<br>46056742	UCHTI<br>CMSSB
HLA	PE	BD	558570	BB7.2
CD11b	PE-CF594	BD	562399	ICRF44
CD15	PE-Cy7	BD	560827	HI98
CD123	BV711	BD	563161	9F5
CD45	APC-R700	BD	566041	HI30
CD14	BUV395	BD	563561	MFP9
CD19	BUV496	BD	564656	SJ25C-1

B)

Markers	Fluorochrome	Vendor	Catalog no.	Clone
LAG3	APC-R700	BD	565774	T47-530
Live_Dead	Aqua Yellow	Invitrogen	L34968	-
CCR6	PerCpCy5.5	BD	560621	11A9
CD127	BV605	BD	562662	HIL-7R-M21
CD49b	BV786	BD	742648	AK7
HLA-A2	PE	BD	558570	BB7.2
CD25	PE-Cy7	BD	557741	M-A251
FoxP3	E-flour450	BD	48477742	259D/C7
CD45RA	BUV-395	BD	740315	5H9
CD45RO	BV711	BD	563722	UCHL1
CD4	BUV496	BD	564651	SK3
CD56	BUV805	BD	748608	MY31
CXCR3	PE-Cy5.5	BD	551128	1C6/CXCR3
CD3	BV650	BD	740562	HIT3A
CD8	BUV737	BD	612754	SK1

Table S5. Immune regulatory and effector cells evaluated in this study

Immune cells	Phenotypic markers	Major Cytokines secreted
Tregs(1)	$CD4^{+}CD25^{+}FoxP3^{+}$	IL-10, TGF-b, IL-35
Tr1(2)	$CD4^{+}$ $FoxP3^{-}$ $LAG3^{+}CD49b^{+}$	IL-10, IL-4, IL-17, TGF-b
eMDSCs(3, 4)	$Lin^{-}CD3^{-}HLA\text{-}DR^{-}CD11b^{+}CD33^{+}$	IL-10, TGF-b, GM-CSF, TNF-a
mMDSCs(3)	$Lin^{-}HA\text{-}DR^{-/low}$ $CD14^{+}CD15^{-}$	
PMN-MDSCs(3)	$Lin^{-}HA\text{-}DR^{-/low}CD14^{-}CD15^{+}CD11b^{+}$	
pDCs(5)	$CD19^{-}CD3^{-}CD56^{-}HLA\text{-}DR^{+}CD11c^{-}CD123^{+}$	IL-10, TGF-b, IL-12
mDCs(6)	$CD19^{-}CD3^{-}CD56^{-}HLA\text{-}DR^{+}CD123^{-}$ $CD11c^{+}$	
Bregs(7)	$CD19^{+}CD24^{hi}CD38^{hi}$	IL-10, TGF-b, IL-35
Th1(6)	$CD3^{+}CD4^{+}CD45RO^{+}CXCR3^{+}$	IFN-g, IL-2, TNF-a
Th17(6)	$CD3^{+}CD4^{+}CD45RO^{+}CCR6^{+}$	IL-17

Table S6. Odds ratio (trend of odds and P trend) of engraftment in plasma cytokine concentration in patients with sickle cell disease who received haploidentical HSCT (* P <0.05, ** P <0.01, *** P <0.001)

Cytokines	BSL		PT-Day 30		PT-Day 60		PT-Day 100		PT-Day 180	
	OR (95% CI)	P value	OR (95% CI)	P value	OR (95% CI)	P value	OR (95% CI)	P value	OR (95% CI)	P value
BAFF	4.00 (0.47, 34.16)	0.170	1.25 (0.19, 8.02)	0.814	0.42 (0.05, 3.69)	0.417	2.00 (0.29, 13.89)	0.475	2.50 (0.29, 21.88)	0.391
bNGF	1.00 (0.11, 9.00)	1.000	0.40 (0.04, 3.90)	0.467	1.33 (0.10, 17.82)	0.782	0.40 (0.03, 4.61)	0.408	2.00 (0.09, 44.35)	0.698
Eotaxin	0.17 (0.02, 1.58)	0.075	1.33 (0.19, 9.21)	0.770	0.17 (0.02, 1.89)	0.102	0.50 (0.07, 3.47)	0.475	2.50 (0.29, 21.88)	0.391
FGF	1.00 (0.05, 20.04)	1.000	4.50 (0.31, 64.80)	0.225	.	0.000***	5.00 (0.53, 47.22)	0.118	5.83 (0.53, 64.33)	0.102
G-CSF	.	0.146	7.20 (0.47, 110.73)	0.098	.	0.002**	2.92 (0.39, 21.68)	0.273	14.00 (0.63, 310.45)	0.028*
GM-CSF	1.00 (0.05, 20.04)	1.000	7.20 (0.47, 110.73)	0.098	.	0.000***	5.00 (0.53, 47.22)	0.118	3.75 (0.40, 35.42)	0.215
GROa	1.50 (0.24, 9.32)	0.661	1.20 (0.18, 7.84)	0.849	.	0.010*	...	0.064	.	0.033*
HGF	.	1.000	0.50 (0.06, 4.34)	0.521	0.50 (0.06, 3.88)	0.499	0.21 (0.03, 1.82)	0.120	.	0.571
IFN-a2	.	1.000	0.20 (0.02, 1.89)	0.118	0.80 (0.11, 5.75)	0.824	0.00	0.873	0.89 (0.12, 6.71)	0.909
IFN-g	1.00 (0.05, 20.04)	1.000	.	0.181	.	0.002**	2.50 (0.08, 81.04)	0.251	3.75 (0.40, 35.42)	0.215
IL-10	.	0.067	5.00 (0.53, 47.22)	0.118	.	0.002**	3.20 (0.37, 27.85)	0.265	14.00 (0.63, 310.45)	0.028*
IL-12p70	0.58 (0.07, 4.88)	0.615	2.00 (0.06, 67.16)	0.462	.	0.018*	.	0.039*	.	0.045*
IL-13	0.26 (0.02, 3.55)	0.276	1.50 (0.04, 53.72)	0.697	.	0.052	.	0.039*	0.00	0.482
IL-15	0.33 (0.02, 4.85)	0.586	6.25 (0.37, 105.77)	0.187	1.17 (0.05, 25.49)	0.569	.	0.361	.	0.222
IL-16	1.33 (0.06, 28.66)	0.511	1.00 (0.05, 19.36)	1.000	6.00 (0.23, 159.40)	0.139	0.00	0.336	0.00	0.499
IL-17A	1.00 (0.05, 20.04)	1.000	4.50 (0.31, 64.80)	0.225	.	0.005**	8.00 (0.71, 90.20)	0.045*	10.00 (0.66, 150.53)	0.040*
IL-18	1.00 (0.16, 6.27)	1.000	1.88 (0.28, 12.51)	0.509	0.48 (0.06, 3.68)	0.470	0.03 (0.00, 1.23)	0.004**	0.10 (0.01, 1.51)	0.040*
IL-1b	0.44 (0.03, 6.46)	0.542	7.20 (0.47, 110.73)	0.098	0.00	0.071	.	0.182	5.83 (0.53, 64.33)	0.102
IL-1RA	1.00 (0.05, 20.04)	1.000	.	0.343	.	0.002**	.	0.089	3.75 (0.40, 35.42)	0.215
IL-2	.	1.000	.	0.377	7.50 (0.28, 199.25)	0.120	14.00 (0.48, 409.57)	0.038*	8.00 (0.30, 214.09)	0.123
IL-2RA	2.33 (0.34, 15.96)	0.374	1.50 (0.18, 12.73)	0.708	2.08 (0.27, 15.97)	0.470	0.43 (0.05, 3.54)	0.418	0.75 (0.10, 5.59)	0.778
IL-3	1.33 (0.10, 17.82)	0.764	0.33 (0.03, 3.79)	0.307	0.75 (0.08, 7.38)	0.799	0.27 (0.02, 4.57)	0.352	0.17 (0.01, 2.39)	0.134
IL-4	0.44 (0.03, 6.46)	0.542	7.20 (0.47, 110.73)	0.098	.	0.002**	5.00 (0.53, 47.22)	0.118	5.83 (0.53, 64.33)	0.102
IL-5	1.67 (0.10, 27.22)	1.000	3.00 (0.17, 52.10)	0.602	3.33 (0.16, 69.43)	0.388	2.00 (0.17, 23.70)	0.591	7.50 (0.28, 199.25)	0.151
IL-6	.	0.745	0.83 (0.08, 8.36)	0.955	2.00 (0.06, 67.16)	0.512	2.00 (0.09, 44.35)	0.758	10.00 (0.19, 516.43)	0.130
IL-7	1.00 (0.14, 7.12)	1.000	5.00 (0.19, 132.83)	0.175	.	0.022*	.	0.003**	.	0.025*
IL-8	1.00 (0.11, 9.47)	1.000	.	0.843	0.50 (0.06, 3.88)	0.499	1.17 (0.16, 8.54)	0.879	.	0.149
IL-9	0.44 (0.03, 6.46)	0.542	4.50 (0.31, 64.80)	0.225	.	0.000***	8.00 (0.71, 90.20)	0.045*	5.83 (0.53, 64.33)	0.102
IP10	0.38 (0.05, 3.07)	0.342	.	0.053	0.75 (0.10, 5.59)	0.778	0.19 (0.02, 1.79)	0.105	0.57 (0.07, 4.66)	0.596
LIF	3.75 (0.22, 64.20)	0.394	.	0.862	0.67 (0.03, 13.31)	0.939	1.00 (0.05, 19.36)	1.000	0.67 (0.05, 8.37)	0.726
MCP-1	1.00 (0.11, 9.47)	1.000	0.43 (0.05, 3.54)	0.418	5.83 (0.53, 64.33)	0.102	2.92 (0.39, 21.68)	0.273	6.00 (0.40, 91.02)	0.141
MCSF	1.00 (0.03, 30.62)	1.000	0.88 (0.09, 8.45)	0.908	.	0.336	0.00	0.763	0.25 (0.01, 6.69)	0.232
MIF	0.88 (0.04, 18.35)	0.739	.	0.515	.	0.035*	1.00 (0.04, 22.61)	0.779	.	0.002**
MIG	4.00 (0.47, 34.16)	0.170	.	0.053	0.75 (0.10, 5.59)	0.778	1.17 (0.16, 8.54)	0.879	0.89 (0.12, 6.71)	0.909
MIP-1a	2.25 (0.15, 32.68)	0.542	3.20 (0.37, 27.85)	0.265	.	0.000***	5.00 (0.53, 47.22)	0.118	14.00 0(.63, 310.45)	0.028*
MIP-1b	0.17 (0.01, 2.36)	0.131	0.50 (0.07, 3.47)	0.475	1.50 (0.17, 13.42)	0.715	0.19 (0.02, 1.79)	0.105	0.11 (0.01, 1.94)	0.068
PDGF-BB	2.25 (0.15, 32.68)	0.542	11.25 (0.63, 201.41)	0.038*	.	0.015*	8.00 (0.71, 90.20)	0.045*	10.00 (0.66, 150.53)	0.040*
RANTES	0.44 (0.03, 6.46)	0.542	4.50 (0.31, 64.80)	0.225	1.25 (0.17, 8.98)	0.824	1.17 (0.16, 8.54)	0.879	0.40 (0.05, 3.50)	0.391
SCF	3.33 (0.16, 69.43)	0.349	0.83 (0.08, 8.95)	0.861	1.00 (0.03, 30.62)	0.931	0.25 (0.01, 4.99)	0.393	0.00.	0.170
SCGFb	0.44 (0.07, 2.89)	0.383	0.50 (0.06, 4.34)	0.521	1.25 (0.17, 8.98)	0.824	3.20 (0.37, 27.85)	0.265	0.89 (0.12, 6.71)	0.909
TGF-b1	2.25 (0.15, 32.68)	0.542	11.25 (0.63, 201.41)	0.038*	.	0.005**	2.92 (0.39, 21.68)	0.273	24.00 (0.69, 835.79)	0.010*
TNF-a	1.00 (0.05, 20.04)	1.000	11.25 (0.63, 201.41)	0.038*	.	0.002**	5.00 (0.53, 47.22)	0.118	14.00 (0.63, 310.45)	0.028*
TRAIL	2.33 (0.34, 15.96)	0.374	0.75 (0.11, 5.18)	0.770	0.75 (0.10, 5.59)	0.778	0.12 (0.01, 1.38)	0.043*	0.60 (0.07, 5.15)	0.638
VEGF	0.58 (0.07, 4.88)	0.615	4.67 (0.55, 39.60)	0.120	.	0.002**	18.00 (0.77, 422.51)	0.013*	3.75 (0.40, 35.42)	0.215

BSL, baseline; OR, Odds ratio; CI, confidence interval; PT, post transplantation; for cytokines-refer to abbreviations above

Table S7. Differences in plasma cytokine concentration levels between the engrafted and rejected groups, linear regression results (* P <0.05, ** P <0.01, *** P <0.001)

Cytokines	BSL		PT-Day 30		PT-Day 60		PT-Day 100		PT-Day 180	
	**Coef. (95% CI)**	***P* value**	**Coef. (95% CI)**	***P* value**	**Coef. (95% CI)**	***P* value**	**Coef. (95% CI)**	***P* value**	**Coef. (95% CI)**	***P* value**
BAFF	0.85 (-0.14, 1.85)	0.089	-0.49 (-1.45, 0.46)	0.291	0.02 (-0.42, 0.45)	0.931	-0.12 (-0.85, 0.61)	0.731	0.24 (-0.31, 0.79)	0.366
bNGF	0.19 (-1.54, 1.92)	0.822	-0.85 (-2.56, 0.86)	0.310	-0.03 (-1.90, 1.84)	0.972	-0.98 (-2.63, 0.67)	0.226	-0.31 (-2.22, 1.60)	0.734
Eotaxin	-0.15 (-0.40, 0.09	0.202	-0.08 (-0.47, 0.32)	0.687	-0.12 (-0.41, 0.18)	0.402	-0.13 (-0.51, 0.24)	0.465	0.22 (-0.14, 0.58)	0.221
FGF	-0.08 (-0.33, 0.17	0.505	0.24 (-0.01, 0.48)	0.059	0.34 (0.12, 0.57)	0.006**	0.39 (0.14, 0.65)	0.004**	0.26 (0.03, 0.50)	0.032*
G-CSF	0.01 (-0.34, 0.37	0.939	0.11 (-0.23, 0.46)	0.490	0.38 (0.12, 0.64)	0.007**	0.18 (-0.65, 1.01)	0.660	0.44 (0.11, 0.77)	0.013*
GM-CSF	-0.08 (-0.34, 0.18	0.515	0.16 (-0.02, 0.34)	0.085	0.24 (0.06, 0.42)	0.014*	0.28 (0.06, 0.50)	0.016*	0.19 (-0.06, 0.43)	0.125
GROa	0.45 (-0.19, 1.10	0.157	0.45 (-0.21, 1.10)	0.169	1.89 (0.02, 3.75)	0.048*	1.91 (-0.29, 4.10)	0.085	1.88 (-0.19, 3.95)	0.072
HGF	1.03 (-0.90, 2.96	0.278	0.03 (-0.69, 0.74)	0.934	0.00 (-0.68, 0.68)	0.995	-0.43 (-1.04, 0.18)	0.153	0.65 (-1.76, 3.06)	0.572
IFN-a2	-0.61 (-1.99, 0.78	0.369	-0.18 (-0.54, 0.18)	0.316	0.06 (-0.30, 0.42)	0.718	-0.64 (-2.17, 0.90)	0.396	0.10 (-0.40, 0.59)	0.676
IFN-g	-0.22 (-0.67, 0.24	0.327	2.00 (-0.56, 4.55)	0.118	2.61 (-0.19, 5.41)	0.066	0.33 (-2.48, 3.15)	0.805	0.25 (-0.20, 0.70)	0.249
IL-10	-0.01 (-0.60, 0.57	0.959	0.49 (-0.17, 1.14)	0.134	0.79 (0.16, 1.41)	0.017*	1.32 (0.44, 2.20)	0.006**	1.09 (0.17, 2.02)	0.024*
IL-12p70	0.10 (-0.65, 0.85	0.781	-0.14 (-2.55, 2.27)	0.903	1.33 (-0.94, 3.59)	0.230	1.60 (0.17, 3.04)	0.031*	1.70 (0.06, 3.35)	0.043*
IL-13	-0.08 (-0.46, 0.30	0.672	-0.60 (-3.24, 2.05)	0.640	-0.15 (-2.05, 1.75)	0.867	1.12 (-0.50, 2.73)	0.162	-0.37 (-2.35, 1.61)	0.696
IL-15	-0.26 (-2.82, 2.29	0.830	2.09 (-0.44, 4.62)	0.099	0.72 (-1.57, 3.00)	0.514	0.98 (-0.95, 2.91)	0.299	0.85 (-0.62, 2.33)	0.234
IL-16	0.53 (-2.20, 3.26	0.687	-0.09 (-3.56, 3.39)	0.959	1.38 (-2.15, 4.90)	0.419	-1.90 (-4.40, 0.60)	0.128	-2.01 (-5.09, 1.07)	0.183
IL-17A	-0.10 (-0.50, 0.29	0.598	0.36 (-0.00, 0.72)	0.052	0.47 (0.15, 0.79)	0.007**	0.60 (0.24, 0.96)	0.003**	0.42 (0.05, 0.78)	0.028*
IL-18	-0.10 (-0.64, 0.45	0.715	-0.27 (-1.22, 0.68)	0.554	-0.23 (-0.89, 0.43)	0.477	-0.76 (-1.42, -0.11)	0.025*	-0.71 (-1.27, -0.14)	0.019*
IL-1b	-0.16 (-0.58, 0.25	0.421	0.28 (-0.43, 1.00)	0.416	0.01 (-1.65, 1.67)	0.990	1.10 (-0.29, 2.48)	0.112	0.46 (-0.22, 1.13)	0.168
IL-1RA	-0.24 (-0.81, 0.32	0.378	0.89 (-1.14, 2.92)	0.370	1.10 (0.32, 1.87)	0.009**	1.74 (-0.18, 3.66)	0.072	0.94 (0.15, 1.73)	0.023*
IL-2	0.08 (-1.30, 1.46	0.906	0.72 (-1.54, 2.98)	0.512	2.17 (-0.05, 4.38)	0.054	2.77 (0.81, 4.73)	0.008**	2.17 (-0.12, 4.46)	0.061
IL-2RA	0.53 (-0.15, 1.21	0.120	0.05 (-0.51, 0.61)	0.863	0.01 (-0.61, 0.63)	0.974	-0.53 (-1.17, 0.11)	0.097	-0.20 (-0.90, 0.49)	0.539
IL-3	0.26 (-3.26, 3.79	0.877	-0.76 (-4.50, 2.99)	0.676	-0.60 (-4.83, 3.63)	0.767	-1.36 (-5.05, 2.32)	0.447	-3.18 (-7.30, 0.93)	0.119
IL-4	-0.06 (-0.32, 0.20	0.633	0.26 (0.02, 0.50)	0.037*	0.33 (0.10, 0.56)	0.009**	0.45 (0.16, 0.74)	0.005**	0.38 (0.08, 0.68)	0.017*
IL-5	0.27 (-1.69, 2.23	0.774	0.06 (-2.49, 2.62)	0.960	0.26 (-2.23, 2.76)	0.827	0.74 (-1.65, 3.13)	0.520	1.32 (-1.16, 3.80)	0.274
IL-6	0.39 (-1.38, 2.16	0.647	-0.45 (-2.76, 1.85)	0.684	-0.15 (-2.21, 1.91)	0.878	-0.25 (-2.44, 1.95)	0.816	1.12 (-0.92, 3.17)	0.260
IL-7	-0.10 (-0.66, 0.47	0.717	1.23 (-1.09, 3.54)	0.279	2.88 (0.16, 5.61)	0.039*	4.63 (2.56, 6.70)	0.000***	3.68 (1.32, 6.03)	0.005**
IL-8	-0.11 (-0.81, 0.59	0.744	-0.37 (-1.72, 0.99)	0.577	0.14 (-0.42, 0.69)	0.610	0.53 (-0.31, 1.38)	0.201	1.30 (-0.05, 2.64)	0.057
IL-9	-0.03 (-0.53, 0.47	0.900	0.38 (-0.12, 0.87)	0.128	0.64 (0.12, 1.17)	0.020*	0.88 (0.38, 1.38)	0.002**	0.71 (0.17, 0.25)	0.013*
IP10	-0.12 (-0.82, 0.58	0.716	-0.12 (-0.69, 0.44)	0.649	0.08 (-0.79, 0.96)	0.844	-0.52 (-1.06, 0.02)	0.056	-0.52 (-1.29, 0.26)	0.176
LIF	0.67 (-1.28, 2.63	0.479	-0.74 (-2.46, 0.98)	0.375	-0.34 (-2.45, 1.77)	0.738	-0.62 (-2.60, 1.35)	0.514	-1.27 (-3.80, 1.25)	0.298
MCP-1	-0.11 (-0.50, 0.29	0.568	-0.09 (-0.48, 0.30)	0.619	0.19 (-0.19, 0.56)	0.309	0.29 (-0.06, 0.65)	0.100	0.38 (-0.00, 0.76)	0.051
MCSF	0.36 (-1.11, 1.84	0.612	0.38 (-0.52, 1.27)	0.387	0.82 (-0.54, 2.19)	0.219	-0.71 (-2.03, 0.61)	0.274	-1.06 (-3.08, 0.95)	0.277
MIF	-0.22 (-2.84, 2.41	0.864	-1.52 (-4.05, 1.00)	0.220	0.41 (-2.54, 3.35)	0.773	0.76 (-2.30, 3.82)	0.605	2.64 (-0.20, 5.47)	0.066
MIG	0.53 (-0.16, 1.22	0.123	-0.02 (-1.02, 0.99)	0.968	0.23 (-1.05, 1.50)	0.709	-0.31 (-1.02, 0.40)	0.370	-0.39 (-1.46, 0.68)	0.443
MIP-1a	-0.08 (-0.35, 0.20	0.553	0.17 (-0.17, 0.50)	0.310	0.41 (0.27, 0.56)	0.000***	0.39 (0.14, 0.65)	0.005**	0.25 (0.01, 0.48)	0.039*
MIP-1b	-0.27 (-0.64, 0.10	0.139	-0.30 (-0.83, 0.23)	0.254	0.22 (-0.37, 0.82)	0.438	-0.34 (-0.87, 0.19)	0.196	-0.67 (-1.22, -0.11)	0.022*
PDGF-BB	0.10 (-0.58, 0.77	0.764	0.62 (-0.05, 1.29)	0.066	1.13 (0.43, 1.83)	0.004**	1.15 (0.43, 1.86)	0.004**	0.84 (0.07, 1.60)	0.034*
RANTES	0.08 (-0.09, 0.26	0.332	0.12 (-0.01, 0.25)	0.065	0.12 (-0.17, 0.40)	0.410	0.05 (-0.14, 0.24)	0.584	-0.05 (-0.27, 0.16)	0.609
SCF	0.65 (-1.52, 2.81	0.539	-0.01 (-2.71, 2.70)	0.994	0.10 (-2.17, 2.37)	0.929	-1.27 (-3.74, 1.20)	0.292	-2.24 (-4.98, 0.51)	0.102
SCGFb	-0.03 (-0.60, 0.53	0.903	-0.06 (-0.48, 0.35)	0.750	0.15 (-0.45, 0.74)	0.611	0.05 (-0.39, 0.50)	0.803	-0.22 (-0.74, 0.31)	0.392
TGF-b1	-0.07 (-0.53, 0.39	0.741	0.52 (-0.16, 1.21)	0.125	1.12 (0.55, 1.69)	0.001**	1.06 (0.42, 1.71)	0.003**	0.46 (-0.17, 1.09)	0.139
TNF-a	-0.11 (-0.56, 0.34	0.615	0.29 (-0.09, 0.67)	0.123	0.50 (0.13, 0.88)	0.012*	0.67 (0.28, 1.06)	0.002**	0.48 (0.07, 0.89)	0.026*
TRAIL	0.23 (-0.41, 0.87	0.464	0.35 (-0.71, 1.40)	0.499	0.20 (-0.36, 0.76)	0.459	-0.31 (-0.83, 0.21)	0.226	-0.02 (-0.50, 0.46)	0.944
VEGF	-0.05 (-0.64, 0.53	0.845	0.64 (0.05, 1.23)	0.036*	0.84 (0.30, 1.38)	0.005**	0.88 (0.36, 1.40)	0.002**	0.68 (0.11, 1.24)	0.022*

BSL, baseline; Coef, Coefficient of linear regression; CI, confidence interval; PT, post transplantation; for cytokines-refer to abbreviations above

Table S8. Relationships between the selected cytokines and transplantation outcome, rotated factor pattern

Cytokines	Factor 1	Factor 2
IL-17A	98*	0
IL-10	96*	-8
IL-7	94*	-2
G-CSF	94*	-9
IL-2	93*	-10
MIP-1a	84*	28
VEGF	79*	12
TGFb1	68*	18
GROa	16	73*
IL-18	-34	62*

*, cytokines contribute significantly to each factor

Table S9. Kaplan Meier estimates based on donor myeloid chimerism at each time point

Time point	Survival	Failure	Error	Failed	Left
BSL/0.000	1.0000	0	0	0	20
PT Day30/30.000	0.8500	0.1500	0.0798	3	17
PT Day60/60.000	0.6500	0.3500	0.1067	7	13
PT Day100/100.000	0.5500	0.4500	0.1112	9	11
PT Day180/180.000	0.4500	0.5500	0.1112	11	9

PT, post transplantation

Supplementary Figures

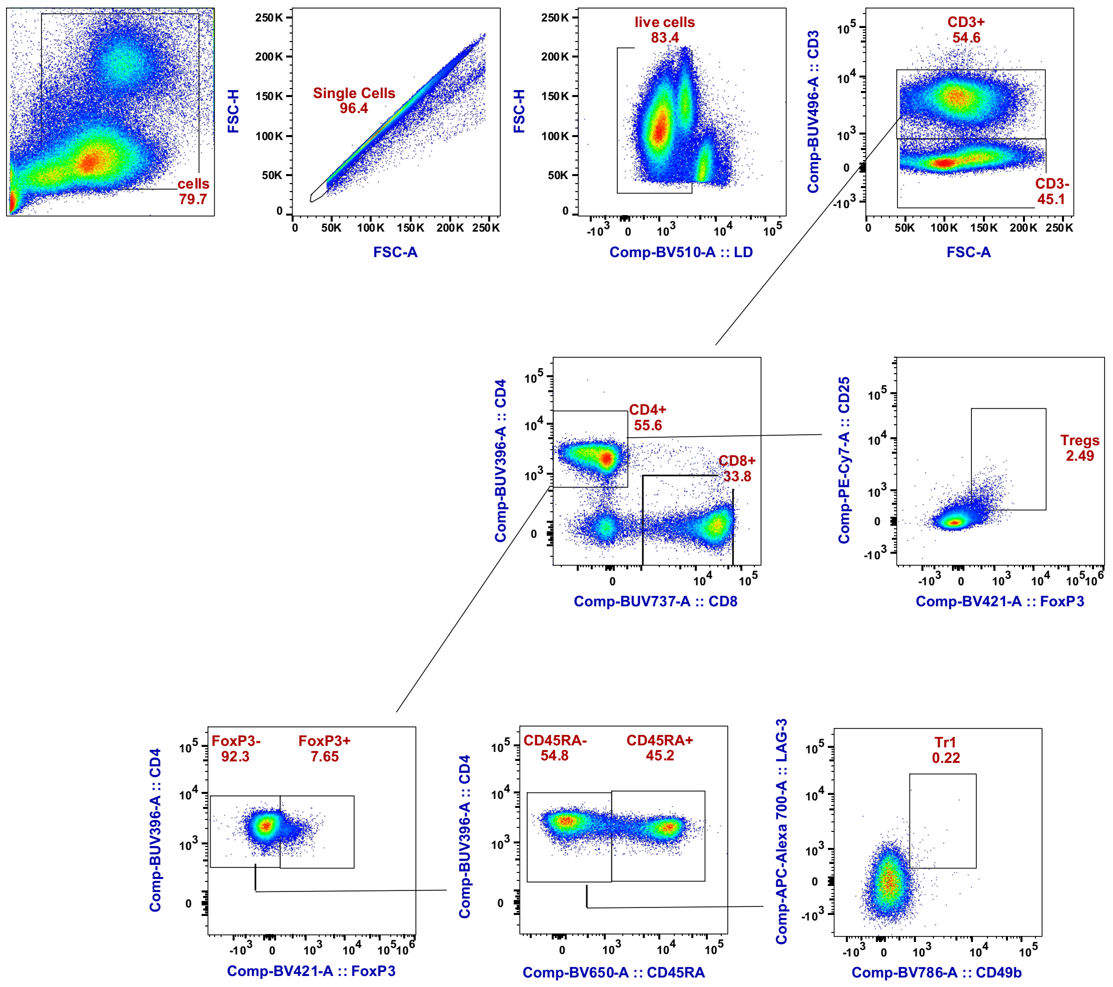

Figure S1

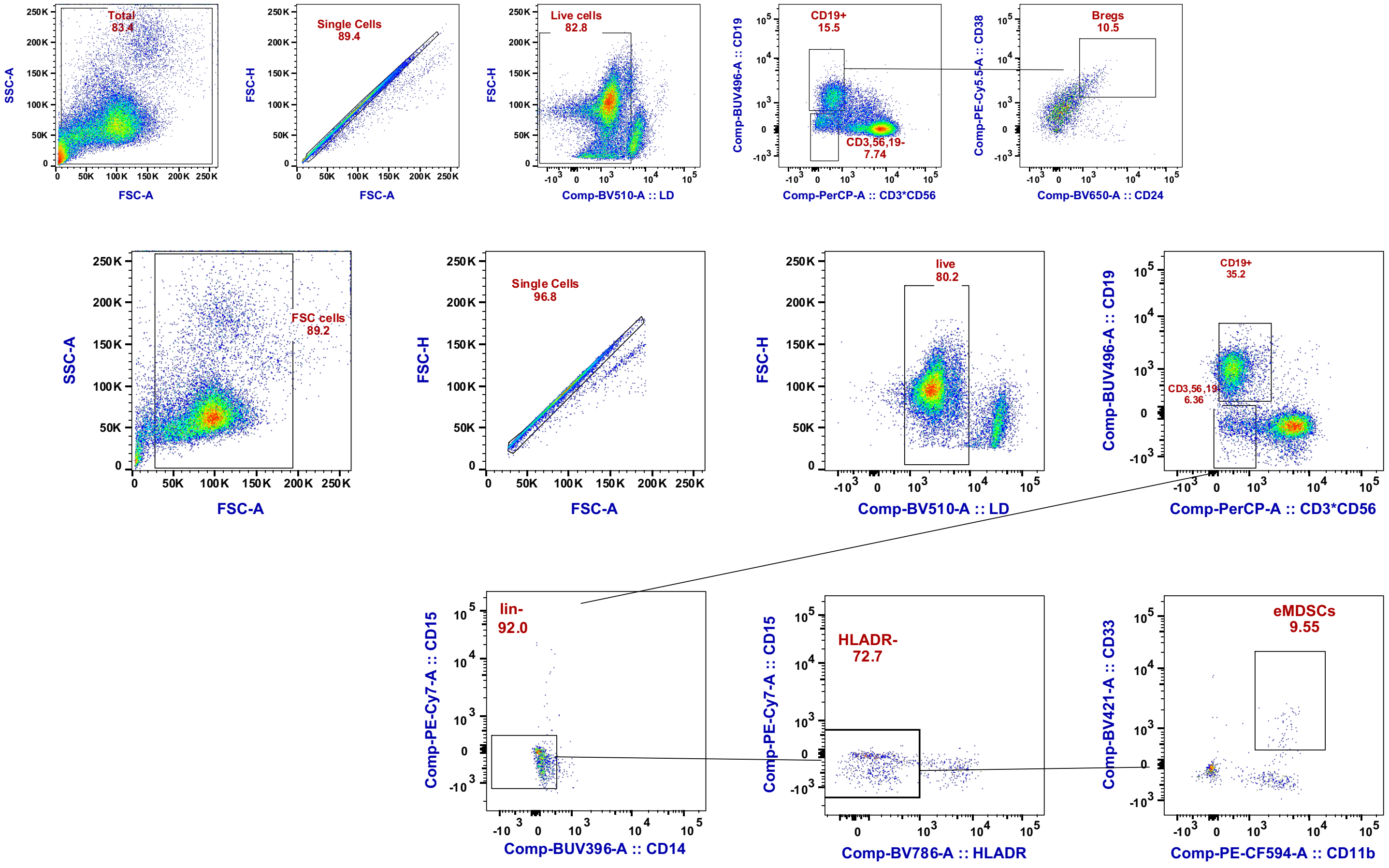

Total
83.4
SSC-A
FSC-A
Single Cells
89.4
FSC-H
Live cells
82.8
Comp-BV510-A :: LD
CD19+
15.5
CD3,56,19-
7.74
Comp-BUV496-A :: CD19
Comp-PerCP-A :: CD3*CD56
Bregs
10.5
Comp-PE-Cy5.5-A :: CD38
Comp-BV650-A :: CD24
FSC cells
89.2
Single Cells
96.8
live
80.2
CD19+
35.2
CD3,56,19-
6.36
lin-
92.0
Comp-PE-Cy7-A :: CD15
Comp-BUV396-A :: CD14
HLADR-
72.7
Comp-BV786-A :: HLADR
eMDSCs
9.55
Comp-BV421-A :: CD33
Comp-PE-CF594-A :: CD11b

Figure S2

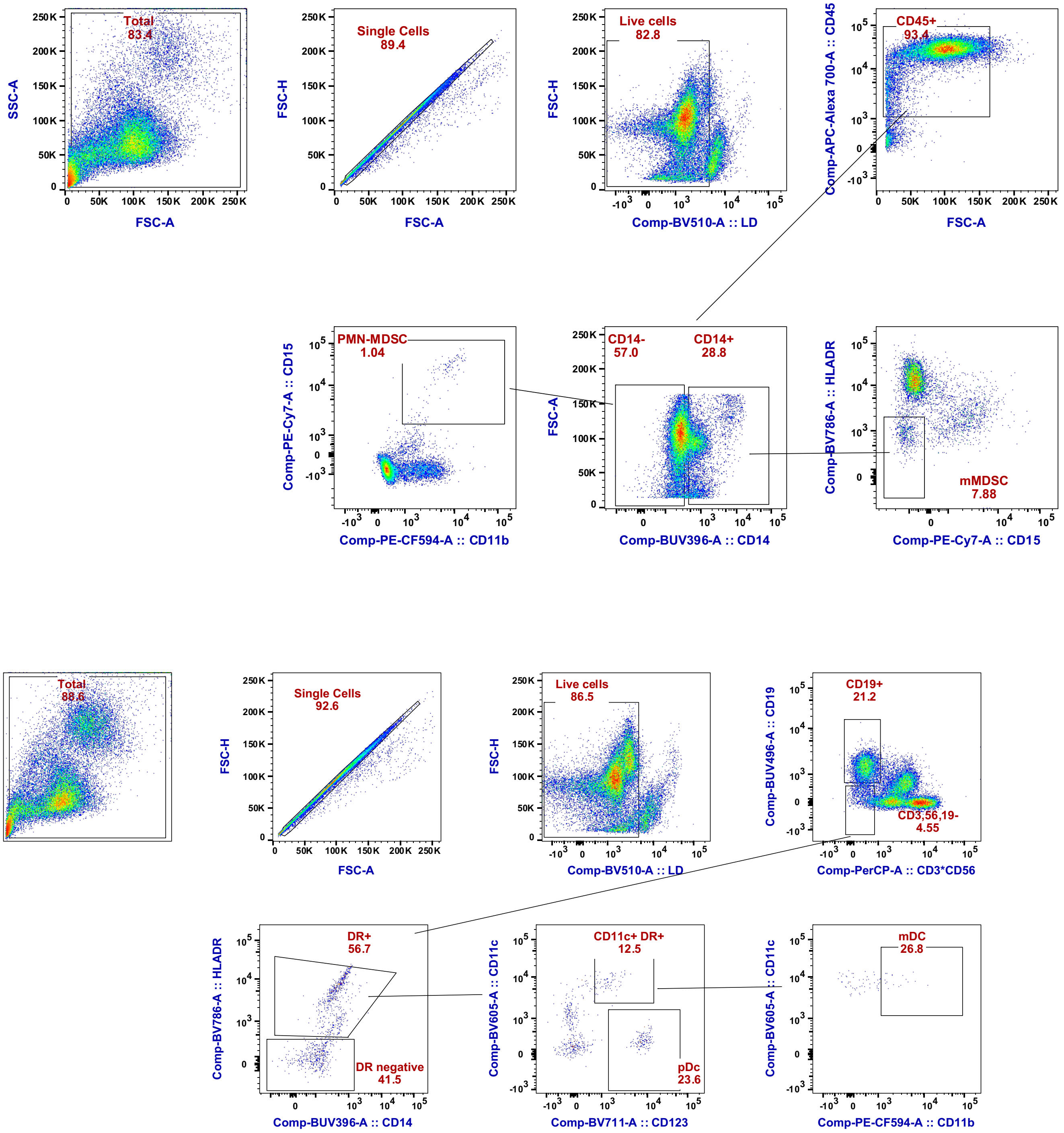
Total
83.4
SSC-A
FSC-A
Single Cells
89.4
FSC-H
FSC-A
Live cells
82.8
FSC-H
Comp-BV510-A :: LD
CD45+
93.4
Comp-APC-Alexa 700-A :: CD45
FSC-A
PMN-MDSC
1.04
Comp-PE-Cy7-A :: CD15
Comp-PE-CF594-A :: CD11b
CD14-
57.0
CD14+
28.8
FSC-A
Comp-BUV396-A :: CD14
Comp-BV786-A :: HLADR
mMDSC
7.88
Comp-PE-Cy7-A :: CD15
Total
88.6
Single Cells
92.6
FSC-H
FSC-A
Live cells
86.5
FSC-H
Comp-BV510-A :: LD
CD19+
21.2
Comp-BUV496-A :: CD19
CD3,56,19-
4.55
Comp-PerCP-A :: CD3*CD56
DR+
56.7
DR negative
41.5
Comp-BV786-A :: HLADR
Comp-BUV396-A :: CD14
CD11c+ DR+
12.5
pDc
23.6
Comp-BV605-A :: CD11c
Comp-BV711-A :: CD123
mDC
26.8
Comp-BV605-A :: CD11c
Comp-PE-CF594-A :: CD11b

Figure S3

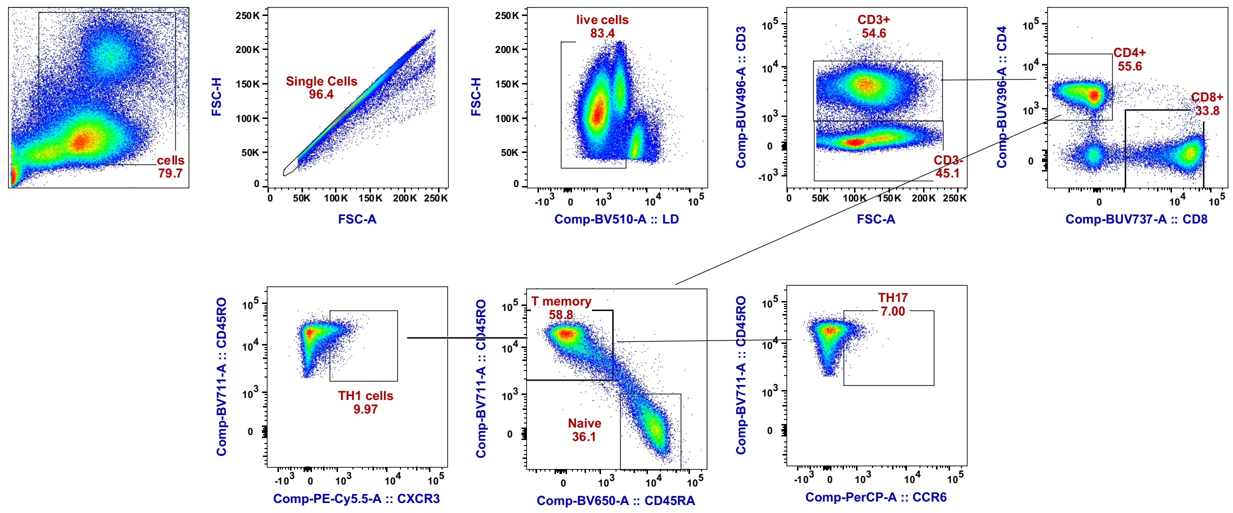
cells
79.7
FSC-H
Single Cells
96.4
FSC-A
live cells
83.4
Comp-BV510-A :: LD
Comp-BUV496-A :: CD3
CD3+
54.6
CD3-
45.1
Comp-BUV396-A :: CD4
CD4+
55.6
CD8+
33.8
Comp-BUV737-A :: CD8
Comp-BV711-A :: CD45RO
TH1 cells
9.97
Comp-PE-Cy5.5-A :: CXCR3
T memory
58.8
Naive
36.1
Comp-BV650-A :: CD45RA
TH17
7.00
Comp-PerCP-A :: CCR6

Figure S4

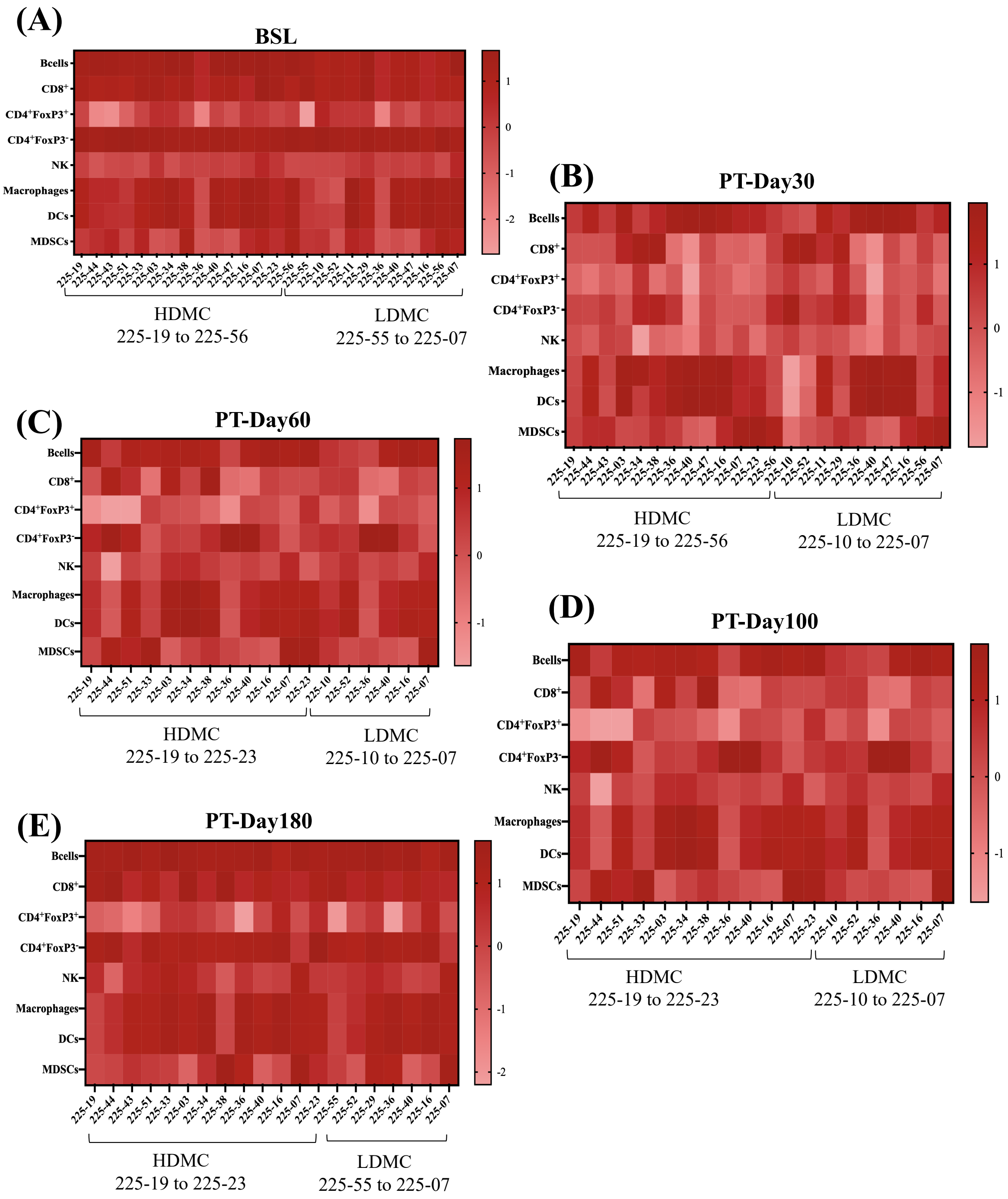
(A)
BSL
Bcells
CD8+
CD4+FoxP3+
CD4+FoxP3-
NK
Macrophages
DCs
MDSCs
HDMC
225-19 to 225-56
LDMC
225-55 to 225-07
(B)
PT-Day30
HDMC
225-19 to 225-56
LDMC
225-10 to 225-07
(C)
PT-Day60
HDMC
225-19 to 225-23
LDMC
225-10 to 225-07
(D)
PT-Day100
HDMC
225-19 to 225-23
LDMC
225-10 to 225-07
(E)
PT-Day180
HDMC
225-19 to 225-23
LDMC
225-55 to 225-07

Figure S5

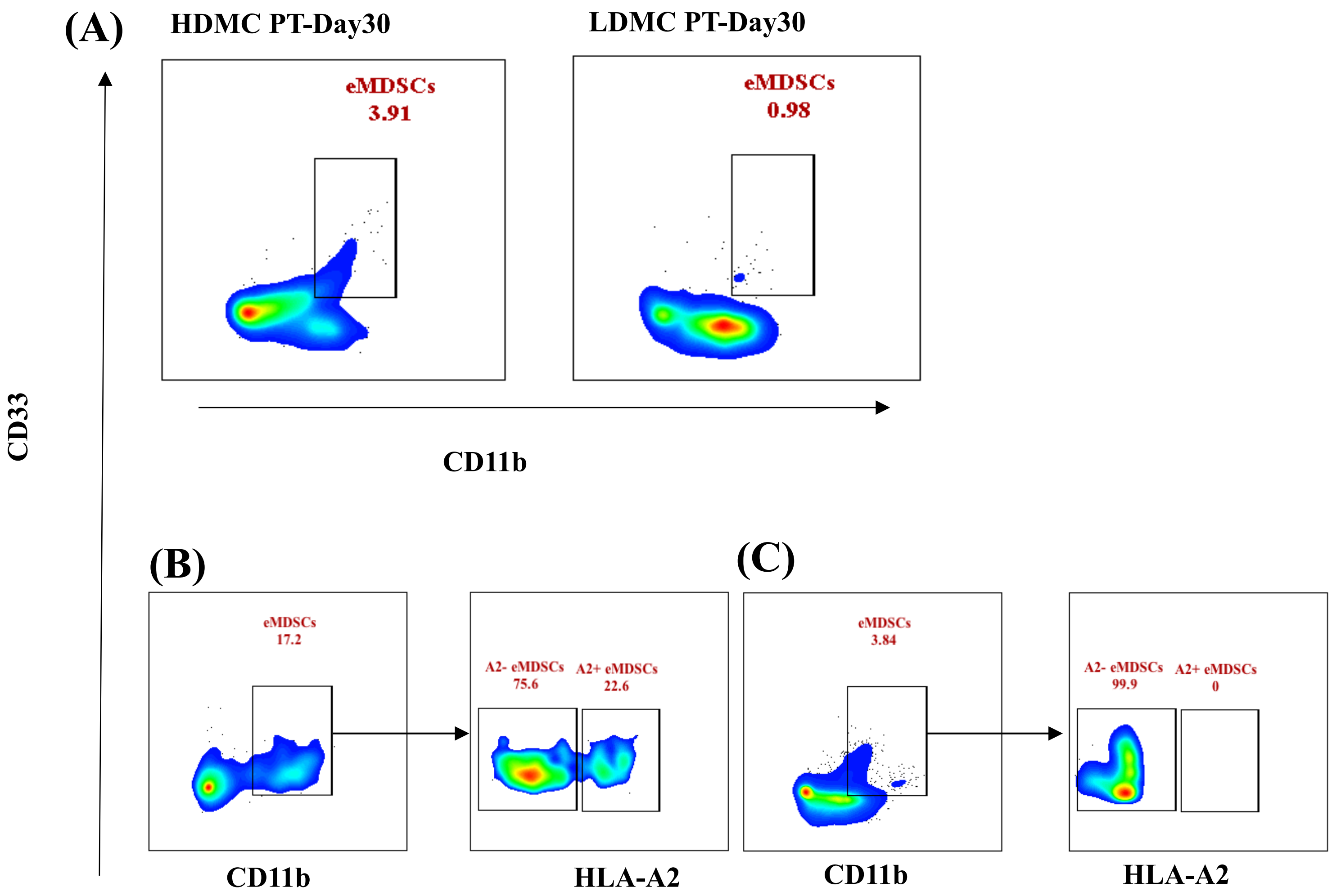
(A)
HDMC PT-Day30
LDMC PT-Day30
eMDSCs
3.91
eMDSCs
0.98
CD33
CD11b
(B)
(C)
eMDSCs
17.2
A2- eMDSCs
75.6
A2+ eMDSCs
22.6
eMDSCs
3.84
A2- eMDSCs
99.9
A2+ eMDSCs
0
CD11b
HLA-A2
CD11b
HLA-A2

Figure S6

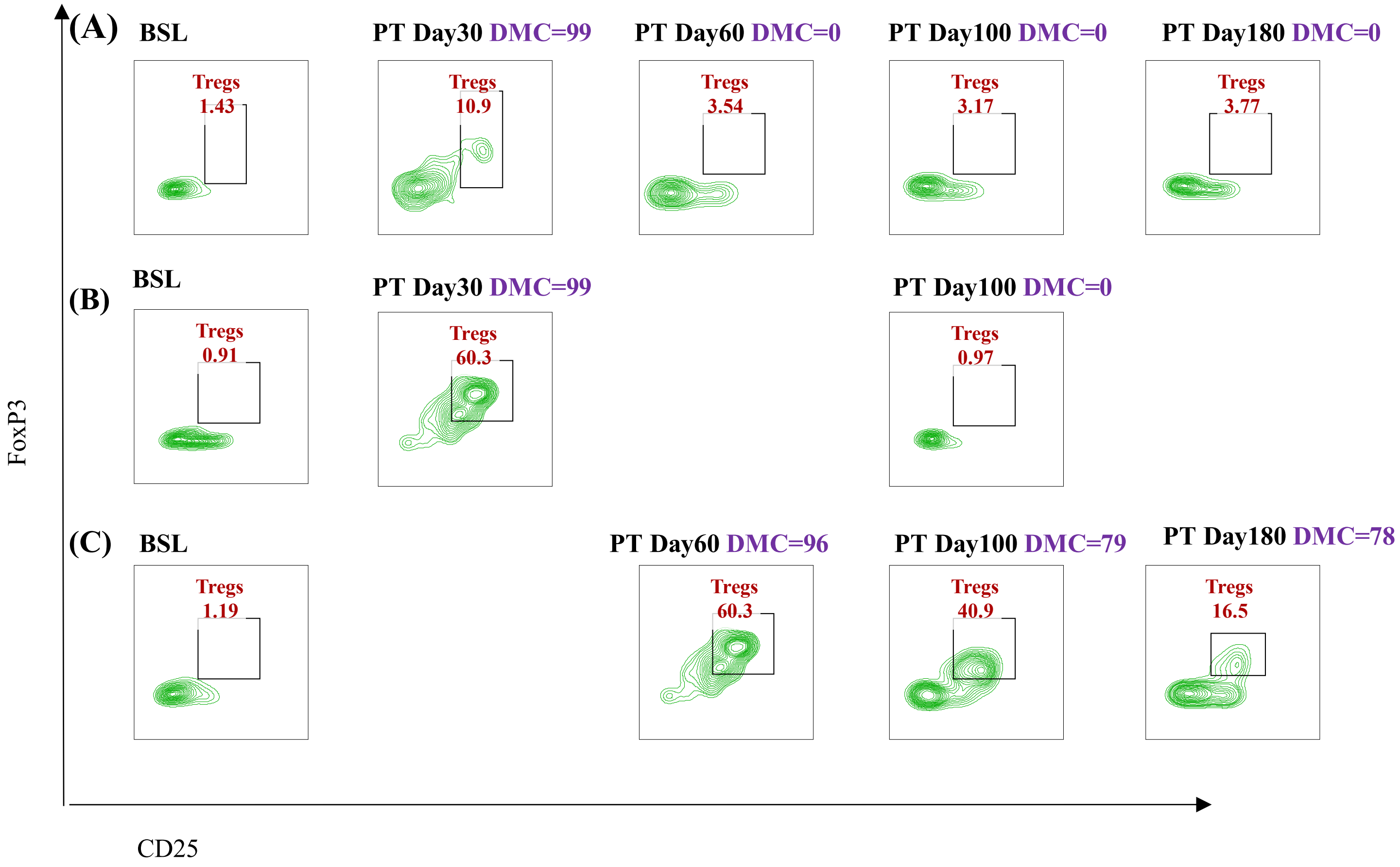
(A)
BSL
Tregs
1.43
PT Day30 DMC=99
Tregs
10.9
PT Day60 DMC=0
Tregs
3.54
PT Day100 DMC=0
Tregs
3.17
PT Day180 DMC=0
Tregs
3.77
(B)
BSL
Tregs
0.91
PT Day30 DMC=99
Tregs
60.3
PT Day100 DMC=0
Tregs
0.97
(C)
BSL
Tregs
1.19
PT Day60 DMC=96
Tregs
60.3
PT Day100 DMC=79
Tregs
40.9
PT Day180 DMC=78
Tregs
16.5
FoxP3
CD25

Figure S7

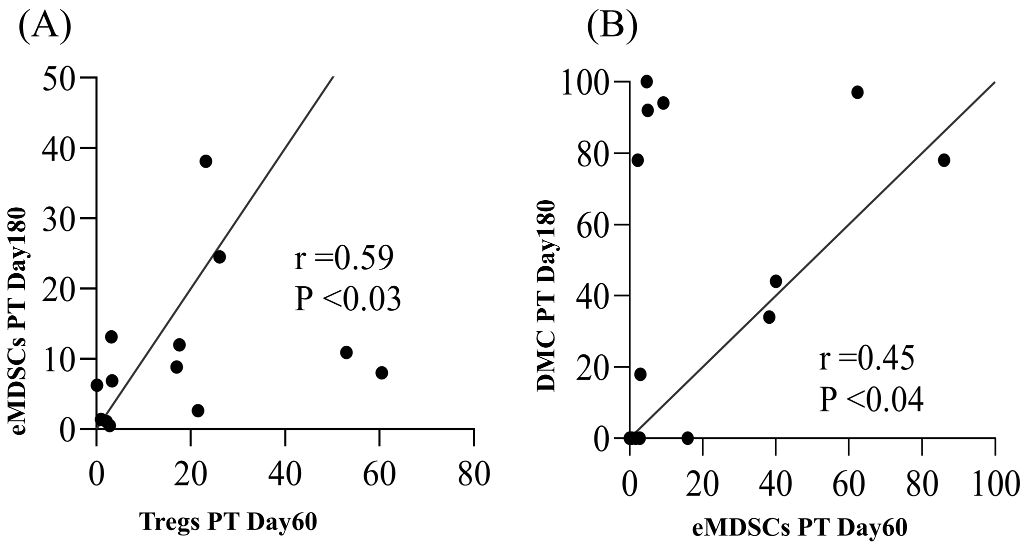
(A)
eMDSCs PT Day180
Tregs PT Day60
r =0.59
P <0.03
(B)
DMC PT Day180
eMDSCs PT Day60
r =0.45
P <0.04

Figure S8

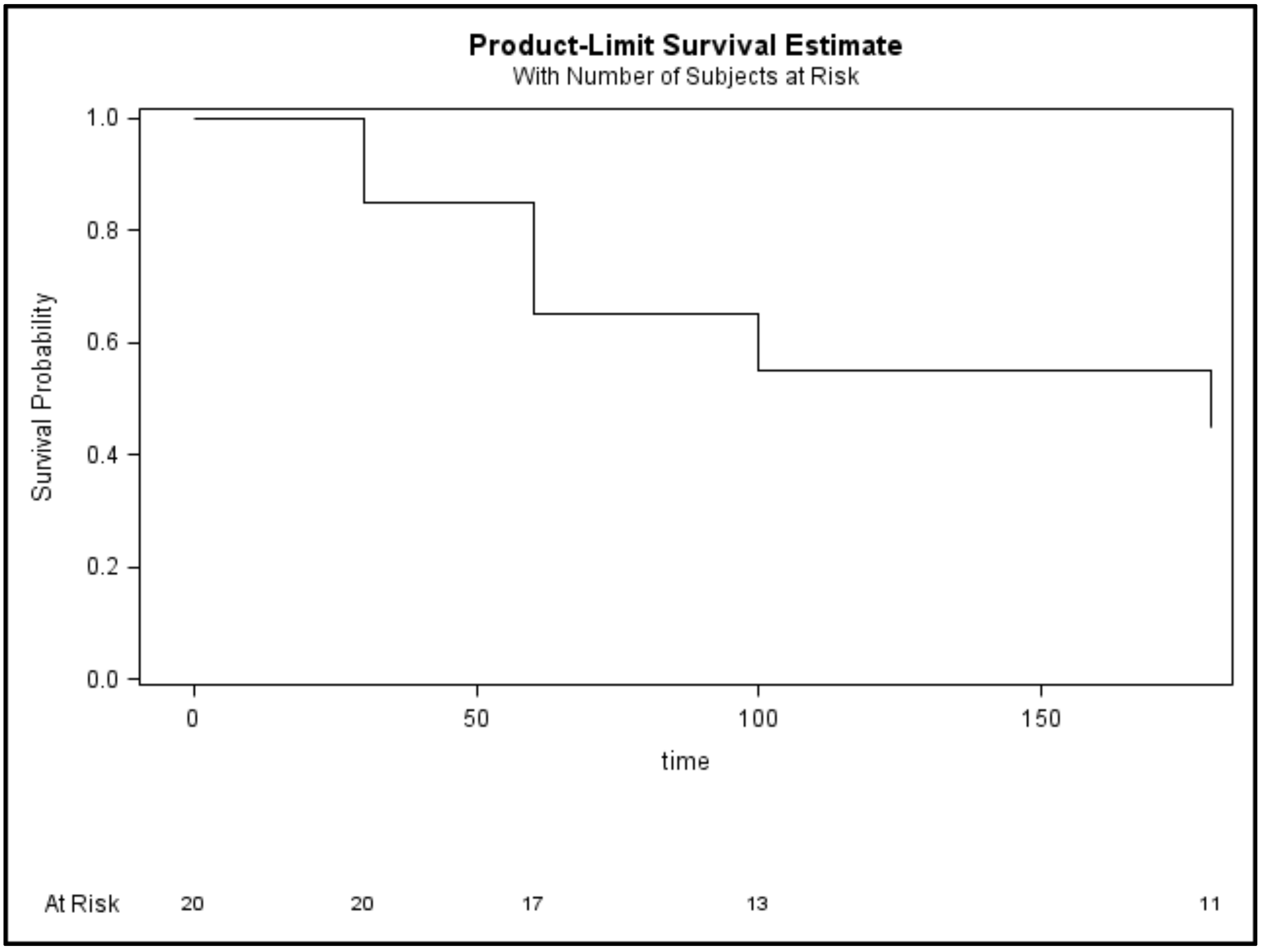
Product-Limit Survival Estimate
With Number of Subjects at Risk
Survival Probability
1.0
0.8
0.6
0.4
0.2
0.0
0
50
100
150
time
At Risk
20
20
17
13
11

Figure S9

Supplementary References

1. Sakaguchi S, Miyara M, Costantino CM, Hafler DA. FOXP3+ regulatory T cells in the human immune system. *Nat Rev Immunol* (2010) 10(7):490-500. Epub 2010/06/19. doi: 10.1038/nri2785. PubMed PMID: 20559327.
2. Gagliani N, Magnani CF, Huber S, Gianolini ME, Pala M, Licona-Limon P, et al. Coexpression of CD49b and LAG-3 identifies human and mouse T regulatory type 1 cells. *Nat Med* (2013) 19(6):739-46. Epub 2013/04/30. doi: 10.1038/nm.3179. PubMed PMID: 23624599.
3. Bronte V, Brandau S, Chen SH, Colombo MP, Frey AB, Greten TF, et al. Recommendations for myeloid-derived suppressor cell nomenclature and characterization standards. *Nat Commun* (2016) 7:12150. Epub 2016/07/07. doi: 10.1038/ncomms12150. PubMed PMID: 27381735; PubMed Central PMCID: PMCPMC4935811.
4. Mandruzzato S, Brandau S, Britten CM, Bronte V, Damuzzo V, Gouttefangeas C, et al. Toward harmonized phenotyping of human myeloid-derived suppressor cells by flow cytometry: results from an interim study. *Cancer Immunol Immunother* (2016) 65(2):161-9. Epub 2016/01/06. doi: 10.1007/s00262-015-1782-5. PubMed PMID: 26728481; PubMed Central PMCID: PMCPMC4726716.
5. Su RJ, Green R, Chen M. Enumeration of bone marrow plasmacytoid dendritic cells by multiparameter flow cytometry as a prognostic marker following allogeneic hematopoietic stem cell transplantation. *Blood Cells Mol Dis* (2018) 69:107-12. Epub 2017/11/16. doi: 10.1016/j.bcmd.2017.10.004. PubMed PMID: 29137845.
6. Biancotto A, Fuchs JC, Williams A, Dagur PK, McCoy JP, Jr. High dimensional flow cytometry for comprehensive leukocyte immunophenotyping (CLIP) in translational research. *J Immunol Methods* (2011) 363(2):245-61. Epub 2010/07/06. doi: 10.1016/j.jim.2010.06.010. PubMed PMID: 20600079; PubMed Central PMCID: PMCPMC2990781.
7. Blair PA, Norena LY, Flores-Borja F, Rawlings DJ, Isenberg DA, Ehrenstein MR, et al. CD19(+)CD24(hi)CD38(hi) B cells exhibit regulatory capacity in healthy individuals but are functionally impaired in systemic Lupus Erythematosus patients. *Immunity* (2010) 32(1):129-40. Epub 2010/01/19. doi: 10.1016/j.immuni.2009.11.009. PubMed PMID: 20079667.
